# Septin multimer autoantibodies in severe motor neuropathy mimicking lower motor neuron disease

**DOI:** 10.1093/brain/awag183

**Published:** 2026-06-08

**Authors:** Friederike A Arlt, Ramona Miske, Luise Appeltshauser, Viktoria Zinnow, Kathrin Borowski, Werner Stenzel, Helena Radbruch, Andreas Meisel, Klemens Ruprecht, Matthias Endres, Igor Blau, Marieluise Kirchner, Philipp Mertins, Elisa Sanchez-Sendin, Stephanie Wernick, Hannah Pressler, Frauke Stascheit, Janis Linke, Sophie Hümmert, Hauke B Werner, Esravila A Wibisono, Kathrin Doppler, Lars Komorowski, Elias T Spiliotis, Divyanshu Dubey, Anastasia Zeckeridou, Sean J Pittock, John R Mills, Andrew McKeon, Madeleine Scharf, Harald Prüss

**Affiliations:** Department of Laboratory Medicine and Pathology, Mayo Clinic, Rochester, MN 55901, USA; Department of Neurology and Experimental Neurology, Charité-Universitätsmedizin Berlin, corporate member of Freie Universität and Humboldt-Universität zu Berlin, Berlin 10117, Germany; German Center for Neurodegenerative Diseases (DZNE) Berlin, Berlin 10117, Germany; Institute for Experimental Immunology, affiliated with EUROIMMUN Medizinische Labordiagnostika AG, Lübeck 23560, Germany; Department of Neurology and Experimental Neurology, Charité-Universitätsmedizin Berlin, corporate member of Freie Universität and Humboldt-Universität zu Berlin, Berlin 10117, Germany; German Center for Neurodegenerative Diseases (DZNE) Berlin, Berlin 10117, Germany; Department of Neurology, University Hospital Würzburg, Würzburg 97080, Germany; Department of Neurology and Experimental Neurology, Charité-Universitätsmedizin Berlin, corporate member of Freie Universität and Humboldt-Universität zu Berlin, Berlin 10117, Germany; German Center for Neurodegenerative Diseases (DZNE) Berlin, Berlin 10117, Germany; Clinical Immunological Laboratory Prof.h.c. (RCH) Dr.med. Winfried Stöcker, Lübeck 23627, Germany; Department of Neuropathology, Charité-Universitätsmedizin Berlin, corporate member of Freie Universität and Humboldt-Universität zu Berlin, Berlin 10117, Germany; Department of Neuropathology, Charité-Universitätsmedizin Berlin, corporate member of Freie Universität and Humboldt-Universität zu Berlin, Berlin 10117, Germany; Department of Neurology and Experimental Neurology, Charité-Universitätsmedizin Berlin, corporate member of Freie Universität and Humboldt-Universität zu Berlin, Berlin 10117, Germany; Center for Stroke Research Berlin, Neuroscience Clinical Research Center, Charité-Universitätsmedizin Berlin, corporate member of Freie Universität and Humboldt-Universität zu Berlin, Berlin 10117, Germany; Neuroscience Clinical Research Center, Charité-Universitätsmedizin Berlin, corporate member of Freie Universität and Humboldt-Universität zu Berlin, Berlin 10117, Germany; Division of Neuroimmunology, Department of Neurology, University of Heidelberg, Heidelberg 69120, Germany; Department of Neurology and Experimental Neurology, Charité-Universitätsmedizin Berlin, corporate member of Freie Universität and Humboldt-Universität zu Berlin, Berlin 10117, Germany; German Center for Neurodegenerative Diseases (DZNE) Berlin, Berlin 10117, Germany; Center for Stroke Research Berlin, Neuroscience Clinical Research Center, Charité-Universitätsmedizin Berlin, corporate member of Freie Universität and Humboldt-Universität zu Berlin, Berlin 10117, Germany; German Centre for Cardiovascular Research (DZHK), partner site Berlin, Berlin 10785, Germany; German Center for Mental Health (DZPG), partner site Berlin, Berlin 10117, Germany; Department of Oncology and Hamatology, Charité-Universitätsmedizin Berlin, corporate member of Freie Universität and Humboldt-Universität zu Berlin, Berlin 10117, Germany; Core Unit Proteomics, Berlin Institute of Health at Charité—Universitätsmedizin Berlin and Max Delbrück Center for Molecular Medicine, Berlin 10178, Germany; Core Unit Proteomics, Berlin Institute of Health at Charité—Universitätsmedizin Berlin and Max Delbrück Center for Molecular Medicine, Berlin 10178, Germany; Proteomics Platform, Max Delbrück Center for Molecular Medicine, Berlin 13125, Germany; Department of Neurology and Experimental Neurology, Charité-Universitätsmedizin Berlin, corporate member of Freie Universität and Humboldt-Universität zu Berlin, Berlin 10117, Germany; German Center for Neurodegenerative Diseases (DZNE) Berlin, Berlin 10117, Germany; Department of Neurology and Experimental Neurology, Charité-Universitätsmedizin Berlin, corporate member of Freie Universität and Humboldt-Universität zu Berlin, Berlin 10117, Germany; German Center for Neurodegenerative Diseases (DZNE) Berlin, Berlin 10117, Germany; Department of Neurology and Experimental Neurology, Charité-Universitätsmedizin Berlin, corporate member of Freie Universität and Humboldt-Universität zu Berlin, Berlin 10117, Germany; German Center for Neurodegenerative Diseases (DZNE) Berlin, Berlin 10117, Germany; Department of Neurology and Experimental Neurology, Charité-Universitätsmedizin Berlin, corporate member of Freie Universität and Humboldt-Universität zu Berlin, Berlin 10117, Germany; Department of Neurology, University Hospital Würzburg, Würzburg 97080, Germany; Rudolf Virchow Center, Center for Integrative and Translational Bioimaging, Julius-Maximilians-Universität Würzburg (JMU), Würzburg 97080, Germany; Department of Neurogenetics, Max Planck Institute for Multidisciplinary Sciences, Göttingen 37077, Germany; Department of Neurogenetics, Max Planck Institute for Multidisciplinary Sciences, Göttingen 37077, Germany; Faculty for Biology and Psychology, University of Göttingen, Göttingen 37073, Germany; Internal Medicine, Saint Mary's Hospital, Waterbury, CT 06706, USA; Department of Neurology, University Hospital Würzburg, Würzburg 97080, Germany; Institute for Experimental Immunology, affiliated with EUROIMMUN Medizinische Labordiagnostika AG, Lübeck 23560, Germany; Department of Cell Biology, University of Virginia School of Medicine, Charlottesville, VA 22903, USA; Department of Laboratory Medicine and Pathology, Mayo Clinic, Rochester, MN 55901, USA; Department of Neurology, Center MS and Autoimmune Neurology, Mayo Clinic, Rochester, MN 55901, USA; Department of Laboratory Medicine and Pathology, Mayo Clinic, Rochester, MN 55901, USA; Department of Neurology, Center MS and Autoimmune Neurology, Mayo Clinic, Rochester, MN 55901, USA; Department of Laboratory Medicine and Pathology, Mayo Clinic, Rochester, MN 55901, USA; Department of Neurology, Center MS and Autoimmune Neurology, Mayo Clinic, Rochester, MN 55901, USA; Department of Laboratory Medicine and Pathology, Mayo Clinic, Rochester, MN 55901, USA; Department of Laboratory Medicine and Pathology, Mayo Clinic, Rochester, MN 55901, USA; Department of Neurology, Center MS and Autoimmune Neurology, Mayo Clinic, Rochester, MN 55901, USA; Institute for Experimental Immunology, affiliated with EUROIMMUN Medizinische Labordiagnostika AG, Lübeck 23560, Germany; Department of Neurology and Experimental Neurology, Charité-Universitätsmedizin Berlin, corporate member of Freie Universität and Humboldt-Universität zu Berlin, Berlin 10117, Germany; German Center for Neurodegenerative Diseases (DZNE) Berlin, Berlin 10117, Germany

**Keywords:** motor neuron autoimmunity, autoantibody discovery, teased fibre assay, peripheral nerve inflammation

## Abstract

Severe neuropathies with predominant involvement of motor fibres can resemble lower motor neuron disease (LMND) phenotypes. Given the fatal prognosis of LMND, identifying underlying autoimmune syndromes is crucial to provide treatment options to patients.

We investigated a novel autoantibody binding pattern observed on murine teased sciatic nerve fibres. Target antigens were identified using immunoprecipitation combined with mass spectrometry. Target specificity of these autoantibodies was validated in cell-based assays, neutralization assays and knock-out models. A retrospective study cohort consisting of different neuropathies (chronic inflammatory demyelinating polyradiculopathy *n* = 86, Guillain–Barré syndrome *n* = 37, multifocal motor neuropathy *n* = 18, diabetic neuropathy *n* = 30, other inflammatory neuropathies *n* = 10), amyotrophic lateral sclerosis (*n* = 50), multiple sclerosis (*n* = 50) and healthy controls (*n* = 50) was negative for septin multimer autoantibodies. Histopathological analysis of skin and the sural nerve including electron microscopy was performed in one seropositive patient, and autoantibody binding was characterized *in vitro*. Extensive immunotherapy was initiated in one patient, with clinical and serological follow-up over 4 years.

Among 3543 total samples tested, three patients (two male, one female)—diagnosed with the LMND variant of amyotrophic lateral sclerosis (aged 65, 72 and 79 years, respectively)—showed a novel and distinct autoantibody binding pattern of indirect immunofluorescence staining on peripheral nerves, targeting Schmidt–Lanterman incisures (SLIs), paranodes and the abaxonal myelin. Target identification and validation revealed septin multimers as autoantibody epitopes. Despite the primarily intracellular location of septins, autoantibody binding was evident in living myelinated dorsal root ganglia, primarily at SLIs (‘incisuropathy’). Septin multimer autoantibodies further initiated complement deposition on fixed and permeabilized cell-based assays. Sural nerve and skin biopsies showed inflammation, myelin and axonal pathology. Extensive immunotherapy in one patient was followed by disease stabilization over 3 years. The other two patients died of rapid disease progression: one of them received no immunotherapy while the other had ineffective treatments with single administrations of intravenous immunoglobulin and rituximab.

Our data suggest that septin multimer autoimmunity occurs in severe motor-predominant neuropathies which can clinically resemble a neurodegenerative LMND. Screening for septin multimer autoantibodies should be considered in patients presenting with this phenotype. Follow-up studies need to determine the direct pathogenicity of septin multimer autoantibodies, their potential as a biomarker of an autoimmune syndrome and responses to immunotherapy in larger cohorts.

## Teaser

Arlt *et al.* describe a novel autoimmune neuropathy (‘incisuropathy’) in three patients initially thought to have an ALS variant. All had antibodies targeting septin multimers at nerve structures. One patient stabilized with immunotherapy, suggesting that some ALS-like syndromes may be potentially treatable autoimmune conditions.

**See Taieb and Devaux (https://doi.org/10.1093/brain/awag233) for a scientific commentary on this article.**

## Introduction

The neurodegenerative lower motor neuron disease (LMND) variant of amyotrophic lateral sclerosis (ALS) is characterized by predominant degeneration of anterior horn cells, leading to progressive denervation, muscle weakness, atrophy and fasciculations, ultimately resulting in respiratory failure. While LMND is driven primarily by neurodegenerative mechanisms rather than immune-mediated injury, its clinical presentation can be mimicked by severe autoimmune neuropathies and neuronopathies with predominant motor fibre involvement, such as motor-predominant chronic inflammatory demyelinating polyradiculopathy (CIDP) and bulbar anti-immunoglobulin-like cell adhesion molecule 5 (IgLON5) disease.^1,2^ In other motor-predominant autoimmune neuropathies such as multifocal motor neuropathy (MMN) and Guillain–Barré syndrome (GBS), autoantibodies against gangliosides have been established as potent disease biomarkers facilitating timely diagnosis and care.^3^ Given the fatal prognosis of LMND, identifying patients with underlying autoimmune disease mimics is crucial to provide treatment options.

Neuronally expressed SEPTIN3, -5 and -7 have been described as targets of autoantibodies in patients with various CNS autoimmune syndromes presenting with cerebellar ataxia, encephalopathy and myelopathy.^4-7^ Septins are ubiquitously expressed, small guanosine triphosphate (GTP)-binding cytoskeletal proteins. Thirteen different human septin isoforms are divided into four groups based on their sequence homologies.^8^ Septin monomers form hetero-hexamers or -octamers which assemble into higher-order complex cytoskeletal structures.^8^ Their functions are cell- and tissue-specific, varying from cell division, polarization and migration to membrane trafficking, subcellular compartmentalization, receptor signalling and cytokinesis.^9,10^ In rodent neuronal cultures, septins function as scaffolds and diffusion barriers coordinating cytoskeletal dynamic proteins, and regulating membrane protein localization, presynaptic fusion and postsynaptic plasticity.^11^

In the myelin of the CNS, SEPTIN2, -4, -7 and -8 assemble into filaments.^12^ These complexes are necessary for myelin integrity as oligodendroglial deficiency of myelin septin filaments causes pathological myelin outfoldings and reduced nerve conduction velocity in mice.^12,13^ In the peripheral nervous system (PNS), however, the relevance of septins for myelin formation and maintenance remains largely unknown.^14^ Further, PNS-reactive autoantibodies targeting septins have not been described to date. Here, we report the identification of a novel type of septin autoantibody, targeting myelin septin multimers in patients with severe inflammatory neuropathies presenting as neurodegenerative LMND.

## Materials and methods

### Study participants, anti-neuronal antibody screening and retrospective cohort screening

Patients included in the study were referred to Charité Berlin or to the Mayo Clinic, Rochester, for anti-neuronal antibody testing. Sera were tested using indirect immunofluorescence assays (IIFA) on mouse brain and nerve tissue as part of the diagnostic workup. At Charité, the brain IIFA is performed on a research basis using an in-house assay with unfixed whole mouse brain sections and sera were diluted at 1:200. The index case was identified in collaboration with EUROIMMUN among 727 consecutive samples tested in 2021 (Cohort 1). Additional cases were identified by applying predefined criteria consisting of: (i) the characteristic brain IIFA staining pattern as used in routine clinical testing at the Neuroimmunology Laboratory of Mayo Clinic Rochester, one of the highest-throughput laboratories worldwide; and (ii) the absence of septin monomer reactivity in cell-based assays (CBA). At the Mayo Clinic, the IIFA is conducted using a validated clinical assay on composite brain tissue slides (#0832-M-BR, Medica Scimedx Corporation) at a serum dilution of 1:240. Two additional cases were identified across two independent cohorts that were sent to EUROIMMUN for septin multimer antibody testing [total amount of samples tested *n* = 2816 (Cohort 2 was previously published^15^)]. The two additionally identified patients were also tested using a peripheral nerve tissue IIFA research-use assay on sciatic nerve teased fibres of mice. For detailed protocols of IIFAs at Charité (brain and sciatic nerve), Mayo Clinic (brain and sciatic nerve) and EUROIMMUN (brain), as well as the flow chart and description of case identifications, refer to the Supplementary material.

Following the identification of three cases with a shared novel autoantibody, we retrospectively screened defined cohorts of patients with ALS fulfilling El-Escorial criteria (*n* = 50), CIDP fulfilling 2021 CIDP criteria^16^ (*n* = 86), GBS (*n* = 37), MMN (*n* = 18), diabetic polyneuropathies (*n* = 30) and patients with other neuropathies (*n* = 10) for septin multimer autoantibodies using brain and nerve tissue IIFA, as well as CBAs expressing septin multimers. Further, healthy controls (*n* = 50) were tested using the research-based assays. Septin multimer autoantibody CBA specificity was further investigated using a disease control cohort of multiple sclerosis patients (*n* = 50).

### Animals

Tissue collection from male C57BL/6 and *Septin7^flox/flox^;Cnp^Cre^* mice followed German animal welfare law (TierSchG §4) and was approved by the *Landesamt für Gesundheit und Soziales* (LaGeSo) in Berlin (approval number T-CH 0009/22), and the *Niedersächsisches Landesamt für Verbraucherschutz und Lebensmittelsicherheit* (LAVES). *Septin7^flox/flox^; Dhh^Cre^* mice (see later) were bred and kept in the mouse facility of the Max Planck Institute for Multidisciplinary Sciences, Göttingen, Germany (MPI-NAT), in accordance with the German animal protection law (TierSchG) and approved by the LAVES. For the procedure of sacrificing vertebrates for tissue preparation, all regulations given in the German animal welfare law (TierSchG §4) were followed. Since sacrificing of rodents including E16 embryos for tissue collection or cell culture is not an experiment on animals according to §7 Abs. 2 Satz 3 TierSchG, no specific ethical review and approval is required for the present work. All procedures were supervised by the animal welfare officers. The animal facilities are registered according to §11 Abs. 1 TierSchG.

### Deletion of *Septin7* in Schwann cells of mice

To delete the *Septin7* gene in Schwann cells, *Septin7^flox/flox^* mice^17^ were interbred with mice expressing *Cre* under control of the *Cnp* promoter^18^ yielding *Septin7^flox/flox^;Cnp^Cre^* mice, also termed *Septin7*-conditional knock-out (cKO), and respective *Septin7^flox/flox^* controls without *Cre*. Genotyping was carried out by genomic PCR.

### Genotyping of *Septin7*-cKO and respective *Septin7^flox/flox^* mice

*Septin7* genotypes were identified using primers 5′-GGTATAGGGG ACTTTGGGG, 5′-CTTTGCACAT ATGACTAAGC and 5′-GCTTCTTTTA TGTAATCCAGG, yielding a 151 bp product for the wild-type allele, a 197 bp product for the unrecombined *Septin7^flox^* allele or a 256 bp product for the recombined *Septin7^flox^* allele. PCR genotyping of the *Cnp* allele was performed with primers 5′-GCCTTCAAAC TGTCCATCTC, 5′-CCCAGCCCTT TTATTACCAC, 5′-CCTGGAAAAT GCTTCTGTCCG and 5′-CAGGGTGTTA TAAGCAATCCC as previously described.^12^ Three mice per genotype (cKO and flox/flox littermates) were used for IIFA experiments on sciatic nerve teased fibres as described later.

### Mouse sciatic nerve teased fibres

Preparations of mouse sciatic nerve teased fibres were conducted as previously described.^19^ In brief, wild-type mouse sciatic nerves were freshly prepared, and immediately fixed in 4% paraformaldehyde (PFA) for 20 min on ice. Epineuriums were removed, and single fibres were teased on glass slides. Slides were air-dried, frozen at −20°C and used within 14 days for IIFAs.

### Indirect immunofluorescence assay

IIFAs on brain tissue performed at Charité, the Mayo Clinic and EUROIMMUN including the neutralization assays, as well as the confocal co-localization studies are described in detail in the Supplementary material. IIFAs on sciatic nerve teased fibres were performed as previously described.^19^ In brief, slides were permeabilized with MeOH for 3 min at −20°C or 0.1% Triton-X for 3 min at room temperature (RT), and washed with PBS. After blocking with blocking solution (10% normal goat serum, 2.5% bovine serum albumin) for 1 h at RT, sera (dilution 1:500) or commercial primary antibodies against septins (SEPTIN2 #ab179436, abcam, 1:50; SEPTIN7 #18991, IBL-Tecan, 1:20) were added overnight at 4°C. After washing, secondary antibodies against human immunoglobulin G (IgG) labelled with Alexa-488 (#109–545-003, Dianova, 1:1000) or against rabbit IgG labelled with Alexa-594 (#111-585-003, JacksonImmunoResearch, 1:500) were added for 2 h at RT. Slides were mounted in mounting medium and kept at 4°C.

### Recombinant expression of septin proteins in HEK293 cells and fixed cell-based assays

The expression of septin monomers (3, 5, 6, 7, 11) and successful multimer expression was described previously^4^ and accordingly performed in this work. Additionally, SEPTIN2 monomers were expressed using a commercially available full-length plasmid (#RC224864, Origene). HEK293 cells were transfected at a confluency of 60%–70% in 50% of regular growth media volume using Polyethylenimine (PEI, #26008, Kyfora Bio) with a 1:5 DNA:PEI ratio. The day after transfection, growth media was added to reach 100% of the final volume. For multimer expression, equal amounts of DNA of each monomer were used to accomplish complex expression (e.g. for SEPTIN3, 5, 6, 7, 11: 0.2 µg of DNA for each monomer leading to a total of 1 µg DNA in the transfection reagent). Forty-eight hours after transfection, cells were fixed with acetone and subjected to immunofluorescence staining using serum and secondary anti-human antibodies. Sera were diluted 1:100 for screening experiments and diluted further to reach end point titres. Sera were diluted in PBS-Tween and added to the transfected and acetone-fixed cells for 30 min at RT. After 5 min of washing with PBS-Tween, cells were incubated with anti-human Pan-IgG secondary antibodies labelled with fluorescein isothiocyanate (FITC) (EUROIMMUN Medizinische LabordiagnostikaAG, undiluted). For IgG subclassification, we used subclass-specific secondary antibodies (Sigma-Aldrich F0767, F4516, F4641, F9890, 1:25).

### Live HEK293 cell-based assays

Recombinant expression of SEPTIN3, 5, 6, 7, 11 multimers was performed as described earlier. Forty-eight hours after transfections, transfected cells and control cells were incubated with PBS-diluted serum (1:20) for 30 min on ice or with a commercial antibody mix consisting of anti-SEPTIN3, -6, -7 and -11 (SEPTIN3 #30146-1AP, Proteintech; SEPTIN6 #12805-AP, Proteintech; SEPTIN7 #18991, IBL-Tecan; SEPTIN11 #14672-1-AP, Proteintech, final dilution 1:20 in PBS). After washing with PBS, cells were stained with secondary anti-human or anti-rabbit Pan-IgG antibodies labelled with FITC (#2040-02 Southern biotech, 1:200) or tetramethylrhodamine isothiocyanate (TRITC) (#4050-03, SouthernBiotech, 1:200) for 30 min on ice. Cells were washed with PBS and fixed with MeOH for 3 min at −20°C. Subsequently, cells that were stained with sera were blocked with blocking solution for 1 h at RT. The commercial antibody mix (anti-SEPTIN3, -6, -7 and -11) was added in a dilution of 1:100 in blocking solution at 4°C overnight. After washing with PBS, secondary anti-rabbit IgGs labelled with TRITC (SouthernBiotech) were added in a dilution of 1:200 for 1 h at RT. Cells were resuspended in PBS and imaged within plates with optical dense plastic bottoms on a confocal microscope.

### Complement deposition assay on fixed HEK293 cell-based assays

For the complement deposition assay, acetone-fixed septin multimer (3, 5, 6, 7, 11) overexpressing HEK293 cells were used as cell-surface expression of septin multimers could not be achieved (Supplementary Fig. 6). Patient serum incubation (30 µl, dilution 1:10) was followed by complement (EUROIMMUN, ZF 9000-0002, 25 µl) incubation for 30 min at RT, and a PBS-Tween wash. To visualize binding of human IgGs to septin multimers and deposition of complement, a directly labelled anti-human-C3c-FITC antibody (EUROIMMUN, AF 612-0115, undiluted) and anti-human-IgG-Cy3 antibody (Jackson ImmunoResearch, 1:400) were incubated simultaneously for 30 min at RT. Fluorescent colours of patient IgG and C3c signals were retrospectively adjusted to keep colouring consistent throughout the manuscript.

### Immunoprecipitation coupled to mass spectrometry

Immunoprecipitation coupled to mass spectrometry (IP-MS) was performed as previously described.^20^ In brief, sciatic nerves of wild-type mice were solubilized in lysis buffer. Sera were bound to Dynabeads™ Protein G (#10004D, Invitrogen), and incubated with the cleared lysates. After washing, we performed on-bead digestion with LysC and trypsin, followed by proteomic analyses using liquid chromatography tandem mass spectrometry (LC-MS/MS). The raw data were processed using the MaxQuant software package with human and mouse UniProt databases (HUMAN.2020-06; MOUSE.2019-07). Label-free quantitation intensities were used for statistical analyses. For target identification, group comparisons against the negative control were employed using the Student’s *t*-test, with a significance threshold set at a false discovery rate (FDR) of 5%.

### Binding assays on living myelinated dorsal root ganglion cultures

Murine myelinating organotypic dorsal root ganglion (DRG) explant co-cultures were cultured and assessed as previously described.^21^ Cells were cultured for 20 days to achieve compact myelination including Schmidt–Lanterman incisures (SLIs) and nodes of Ranvier. They were either fixed and co-stained with serum and commercial antibodies, or living cells were incubated with filtrated serum (1:100) in myelination medium for 2 or 5 days, before fixation and co-staining with anti-human IgG. Immunofluorescence was performed as described previously^21^ using antibodies against SEPTIN7 (1:20), SEPTIN2 (1:100), Neurofascin (#AF3235, R&D Systems, 1:5000) and myelin-associated glycoprotein (MAG; #MAB1567, Merck, 1:1500), along with appropriate secondary antibodies. All experiments were performed twice.

### Immunofluorescence analysis of skin

Dermal punch biopsies from the lateral index finger and the leg were stained with anti-myelin basic protein (MBP; #GTX133108, GeneTex, 1:200) and anti-Neurofascin (R&D Systems, 1:400), and anti-Caspr1 (#ab34151, Abcam) as described previously.^22^

### Morphologic analysis of sural nerve and skin biopsies

Diagnostic conventional histology and enzyme histochemistry reactions [haematoxylin and eosin (H&E), Gömöri trichrome, acid phosphatase, Elastica van Gieson, Kongo red, CD3, CD8, CD31, CD45, CD68, C5b-9, CD20, CD138, neurofilament] were performed on 8-µm thick cryostat sections according to international recommendations and as described previously.^23,24^ Teased fibre preparations from the sural nerve were conducted according to established protocols.^25^

### Electron microscopy of sural nerve biopsy

Nerve tissues were fixed with 2.5% glutaraldehyde in 0.1 M sodium cacodylate buffer, postfixed with 1% osmium tetroxide in 0.05 M sodium cacodylate, dehydrated and embedded in Renlam resin. Uranyl acetate and phosphotungstic acid were used for contrasting; 70 nm ultrathin sections were cut using an ultramicrotome, stretched with xylene vapour, collected onto pioloform-coated slot grids and stained with lead citrate. Transmission electron microscopy was performed using a Zeiss 906 microscope with a 2k CCD camera (TRS).

### Ethics and study approval

The study was conducted in accordance with the Declaration of Helsinki. All participants gave written informed consent for study participation and serum biomarker analyses. The study was approved by the ethics board of Charité (#EA1/258/18) and the Mayo Clinic Rochester (IRB #23-007528 and #21-1297).

### Software and data visualization

Microscope images including maximum projections of *z*-stacks were processed using the FIJI software,^26,27^ Imaris Version 9.9.1 (Bitplane, Belfast, UK). Data visualization was performed in Prism Version 9.4.1 (GraphPad Software, San Diego, CA, USA), Inkscape Version 1.2.1 (Inkscape Project. 2020, https://inkscape.org), Illustrator (Adobe Inc., San José, CA, USA) and using Biorender.com.

## Results

### Patients with suspected LMND exhibit autoantibodies against SLIs, paranodes and abaxonal myelin

The here-reported patients showed a positive autoantibody result on brain and nerve tissue, revealing a previously undescribed binding pattern on teased nerve fibres. The index patient was referred to Charité (among a total of 727 consecutive samples tested in 2021) while the other two patients were found via retrospective screening (total amount of samples tested *n* = 2816). For additional detailed information on the identification of all three cases refer to the flow chart (Supplementary Fig. 1) and the description in the Supplementary material. All three patients (two males, one female), aged 65, 72 and 79 years at symptom onset, presented with fine motor skill impairment, progressive and severe, asymmetric, distally predominant muscle weakness, and atrophy with fasciculations and limb cramps, predominantly affecting the lower limb in Patients 1 and 3, and the upper limb in Patient 2. Disease progression was rapid in Patient 3, moderate in Patient 2 and relatively slow in Patient 1. From symptom onset to requiring a wheelchair ambulance, Patient 1 deteriorated over 3 years, whereas Patient 3 became wheelchair-bound within just 4 months.

Patients 1 and 3 initially complained of sensory symptoms, which were right-dominant distal hypoaesthesia for touch, temperature and pain sensation starting in both legs in Patient 1, and paraesthesia (pins and needle sensation) in both legs and feet in Patient 3. None of the patients had neuropathic pain. Bulbar involvement with dysphagia and intermittent aspirations developed in Patient 1 (late in the disease course) and within months in Patient 3, while information on bulbar function was not available for Patient 2.

Electrophysiological studies in all patients showed distal axonal and motor-predominant neuropathy with accompanying demyelinating features (reduced nerve conduction velocity in one nerve in Patient 1 and prolonged distal motor latencies in two nerves in Patient 3). Sensory nerve conduction studies showed abnormalities in two to three nerves in Patients 1 and 3. Thus, those two patients fulfilled electrodiagnostic criteria^16^ for (possible) multifocal CIDP, but clinical criteria for multifocal CIDP were not met due to the lack of additional suggestive criteria (Patient 1), normal tendon reflexes (Patient 3) and a clinically more probable differential diagnosis. No conduction blocks suggestive of MMN were present. Axonal damage increased over time in Patient 1 where longitudinal data on electrophysiology was available (Supplementary Tables 1 and 3). EMG showed active denervation, and pathological spontaneous activity in the extremities and paravertebral muscles of Patients 1 and 2 (Supplementary Table 2). Spinal cord imaging remained without inflammatory correlates of symptoms in all three patients. CSF status revealed a minimal protein elevation in Patient 2 and Patient 3 while other CSF status parameters were normal. For additional detailed clinical data, refer to Table 1. In all three patients, an axonal-demyelinating neuropathy was initially suspected, and autoimmune aetiology was considered by the treating physicians who initiated testing for autoantibodies. Patients 2 and 3 both showed a low-titre positive VGCC-P/Q (voltage-gated calcium channel P/Q subunit) test result (0.05 nmol/l, reference <0.02 nmol/l) while other autoantibody panel testing remained negative (Supplementary Table 4). Owing to asymmetric and partial upper limb presentation, signs of paravertebral and bulbar denervation, motor predominance with evolving and severe muscle atrophy and moderate to rapid disease progression despite initial trials of IVIg therapies (in Patients 1 and 3), all three patients were ultimately diagnosed with the LMND variant of ALS.

**Table 1:** Clinical data of patients with septin multimer autoantibodies

Patient number, age, gender	Septin multimer CBA titre	Working diagnosis	Clinical presentation	MRI	Electrophysiology	NfL serum NfL CSF CSF status Abs serology	Immunotherapy, disease course, follow-up
#1, 65 years, male	1:320 000	Lower motor neuron disease	Progressive, asymmetric tetraparesis with fasciculations and atrophy (legs > arms), flail leg syndrome. Fine motor impairment of hands. Areflexia of lower limbs. Mild sensory symptoms with initial numbness, paraesthesia in legs (right > left, distal), progress to upper extremities.	Cerebral: normal Spine: no spinal cord atrophy	NCS: axonal > demyelinating, motor > sensory neuropathy. EAN/PNS electrodiagnostic criteria: possible CIDP. EMG: pathological spontaneous activity in upper and lower limb and paravertebrally. MEPs: no sign of affected 1st motor neuron.	23.3 pg/ml (ref. range: < 30.90) 1140 pg/ml (ref. range: < 2500) 1 cell/µl (ref. range: 0–4), protein CSF: 445.4 mg/l (ref. range: 150–450)	Chronic onset, moderately progressive with subacute deteriorations (nadir within 4 years). Extensive immunotherapy (Fig. 6). Stabilization for 4 years.
#2, 72 years, male	1:100 000	Lower motor neuron disease	Progressive, asymmetric tetraparesis with limb cramps and atrophy (arms > legs), flail arm syndrome. Fine motor impairment of hands. Areflexia of upper extremities. No reported sensory symptoms.	Cerebral: normal Spine: no spinal cord atrophy	NCS axonal > demyelinating, motor-sensory neuropathy. EAN/PNS electrodiagnostic criteria: not fulfilled. EMG: pathological spontaneous activity in upper and lower limb and paravertebrally. MEPs: no sign of affected 1st motor neuron.	NfL serum/CSF n.d. 1 cell/µl (reference range: 0–5), protein CSF: 37 mg/dl (ref. range: 0–35) VGCC-P/Q: 0.05 nmol/l^a^	Chronic onset, moderately progressive (nadir within 2 years). No immunotherapy. Died 2 years after onset due to pneumonia with advanced stage motor impairment.
#3, 79 years, female	1:100 000	Lower motor neuron disease	Progressive, asymmetric tetraparesis (legs > arms), limb cramps, bilateral thenar muscle atrophy. Gait abnormalities with frequent falls. Dysphagia, vocal cord paralysis. Reflexes normal in both upper and lower limbs. Mild, symmetric sensory symptoms in distal extremities (paraesthesia, dysaesthesia).	Cerebral: mild atrophy, moderate chronic small vessel ischaemic changes, small old lacunar infarct in right thalamus and right caudate. Spine: multilevel spondylitis.	NCS: upper and lower axonal, motor > sensory neuropathy, left > right. EAN/PNS electrodiagnostic criteria: possible CIDP. EMG: normal. MEPs: not performed.	NfL serum/CSF n.d. 2 cells/mm^3^ (ref. range 0–5), CSF protein 67 mg/dl (ref. range 15–45 ) VGCC-P/Q: 0.05 nmol/l^a^	Subacute onset, rapidly progressive, wheel-chair bound within 4 months. Died 14 months after onset due to infection (abdominal skin abscess/cellulitis) with PEG. Treated with IVIg 2 g/kg over 5 days without reported benefit. Prednisolone taper dose without improvement. One dose of rituximab without reported benefit.

Electrodiagnostic findings of NCS were initially interpreted as carpal tunnel syndrome in Patients 1 and 2. Abs = autoantibodies; CBA = cell-based assay; CIDP = chronic inflammatory demyelinating polyradiculoneuropathy; EAN/PNS = European Academy of Neurology/Peripheral Nerve Society; IVIg = intravenous immunoglobulin; MEPs = motor-evoked potentials; NCS = nerve conduction studies; NfL = neurofilament light chain; n.d. = not done; PEG = percutaneous endoscopic gastrostomy; VGCC-P/Q = voltage-gated calcium channel P/Q subunit.

^a^Staining with a high-positive VGCC-P/Q serum and commercial VGCC-P/Q antibody did not show a similar pattern to Patients 1–3 serum on sciatic nerve teased fibres (Supplementary Fig. 7).

**Figure 1:**
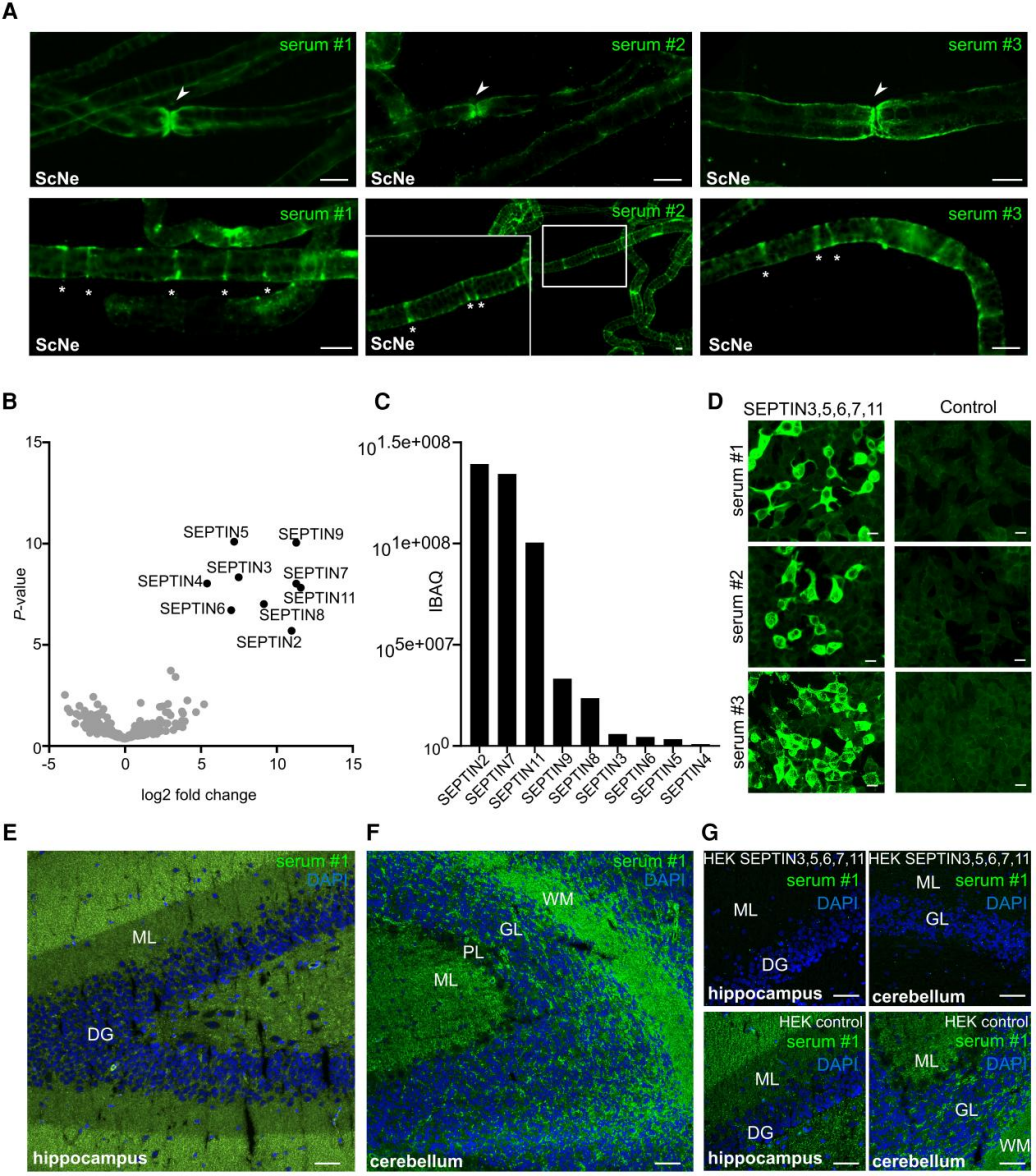
**Autoreactive IgGs target septin multimers.** (**A**) Representative images of indirect immunofluorescence on mouse sciatic nerve teased fibres showed reactivity of Patients 1–3 serum to paranodes and paranodal myelin (arrowhead), as well as Schmidt–Lanterman incisures (asterisk). (**B**) Volcano plot representing significantly enriched proteins (labelled in black) in Patient 1 IgG immunoprecipitation (IP) compared with a negative control; the *x*-axis displays the log2-transformed fold change, and the *y*-axis represents the -log10-transformed *P*-value. (**C**) IBAQs (intensity-based absolute quantification) of mass spectrometry-identified proteins in the IP of Patient 1. (**D**) Cell-based assays with sera of Patients 1–3 showed IgG reactivity against septin multimer overexpressing HEK293 cells, but not against untransfected control HEK293 cells. (**E**–**F**) Representative images of indirect immunofluorescence on unfixed rat brain sections showing IgG reactivity of serum 1 against the molecular layer of the hippocampus (**E**), and the molecular layer and white matter in the cerebellum (**F**). (**G**) Neutralization assays on brain tissue showed full absorbance of the IgG signal after preincubation with septin multimer HEK293 cell extracts, while preincubation with control HEK293 cells did not alter the tissue reactivity. Scale bars = 10 µm (**A** and **D**); 50 µm (**E**–**G**). IgG =immunoglobulin G.

The novel binding pattern of autoantibodies on teased nerve fibres involved the abaxonal myelin, the paranodes and SLIs (Fig. 1A), which was not detected in healthy controls (*n* = 50).

### Autoantibodies target septin multimers in PNS myelin

To identify the autoantibody targets, we used the index patient serum (Patient 1) and applied two parallel strategies: CBAs expressing different neuronal antigens, and an untargeted approach using IP-MS with sciatic nerve lysate. IP-MS revealed binding to several septin proteins (Fig. 1B). Among them, SEPTIN2 and SEPTIN7 had the highest protein abundance (Fig. 1C). Interestingly, different to septin autoantibodies described previously,^4-6^ the sera of all patients showed no or only weak reactivities with several septin monomers (SEPTIN2, -3, -5, -6, -7 or -11, Supplementary Fig. 2A). Overexpression of septins in a multimer (constituted of SEPTIN3, -5, -6, -7, -11), however, showed strong binding of the patients’ autoantibodies (Fig. 1D, serum titre of 1:320 000 in Patient 1 and 1:100 000 in Patients 2 and 3). Septin multimer reactivities in CBA could not be abolished by depletion of one of the septins from the multimer (Supplementary Fig. 2B). Testing of the patients’ sera on protein microarray displaying full-length human proteins showed no binding to single septins, further suggesting that no linear epitope to septin proteins is detected (Supplementary Fig. 2C). Sera also reacted with rat brain sections in the hippocampus and the cerebellum showing a characteristic septin staining pattern^4-6^ (Fig. 1E and F) and additionally showed white matter reactivity (Fig. 1F). Tissue reactivity was abolished by preincubation of patients’ sera with HEK cell extracts expressing SEPTIN3, -5, -6, -7, -11 multimers (Fig. 1G). In contrast, pre-adsorption using SEPTIN3 or -7 alone did not abolish tissue binding (Supplementary Fig. 3A). Further, heat-denatured HEK cell extracts expressing SEPTIN3, -5, -6, -7, -11 multimers did not neutralize tissue binding, and neither did a mixture of HEK cell extracts expressing SEPTIN3, -5, -6, -7, -11 monomers (Supplementary Fig. 3B and C). A bacterially expressed and purified hetero-octamer consisting of SEPTIN2, -6, -7 and -9 was similarly effective in abolishing tissue binding as the HEK cell extracts expressing the SEPTIN3, -5, -6, -7, -11 multimers (Supplementary Fig. 3D). Taken together, these findings confirm that a conformational epitope only present after septin multimer formation is recognized.

**Figure 2:**
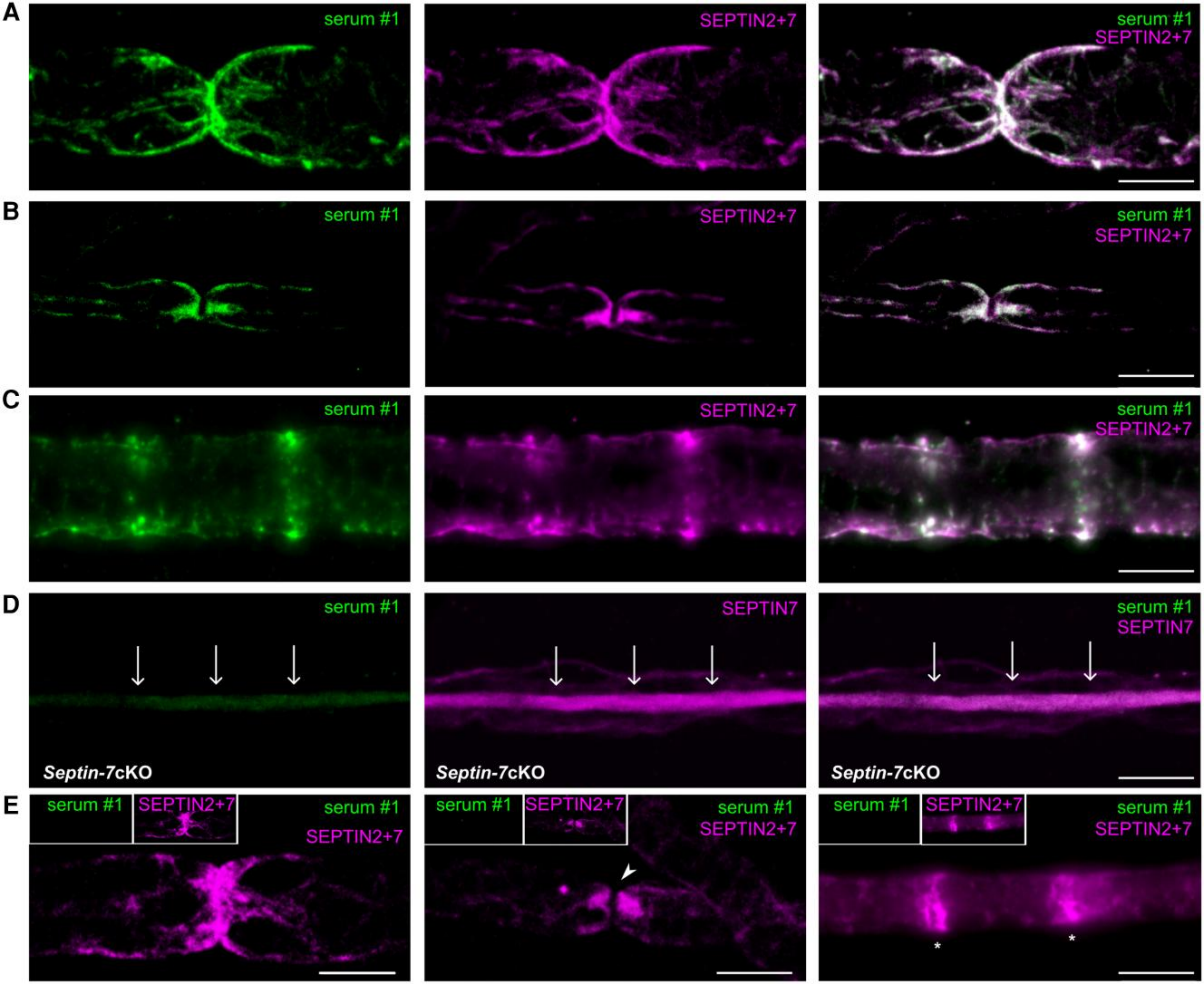
**Septin multimer IgG demonstrate a characteristic binding pattern to PNS myelin.** (**A**–**C**) Representative images of indirect immunofluorescence with serum of Patient 1 and commercial SEPTIN2 and SEPTIN7 antibodies on sciatic nerve teased fibres demonstrated signal overlay at the paranodal myelin of large calibre fibres (**A**), paranodes of small calibre fibres (**B**) and Schmidt–Lanterman incisures (**C**). (**D**) Indirect immunofluorescence of serum 1 on sciatic nerve teased fibres of *Septin7*-conditional knock-out (cKO) mice lacking *Septin7* expression in Schwann cells. The serum demonstrated only weak axonal reactivity, while the commercial anti-SEPTIN7 antibody displayed axonal immunolabelling (arrows) when SEPTIN7 was lacking from myelin. Three animals per genotype were stained per group and showed equal results as represented here. (**E**) Neutralization assays on wild-type (WT) nerves showing complete loss of serum binding upon preincubation with septin multimer HEK293 cell extracts. Sequential staining of non-absorbed commercial anti-SEPTIN2 and SEPTIN7 antibodies (magenta) indicate the presence of SEPTIN2 and -7 patterns. Scale bars = 10 µm (**A**–**E**). IgG = immunoglobulin G; PNS = peripheral nervous system.

Staining patterns of septin autoantibodies have so far not been described on peripheral nerves. Here, serum binding co-localized with anti-SEPTIN2 and anti-SEPTIN7 commercial antibodies at the paranodal myelin of large calibre fibres (Fig. 2A), the paranode of small calibre fibres (Fig. 2B) and the SLIs of myelinated fibres (Fig. 2C). To investigate binding properties to peripheral nerve myelin deficient of septin proteins, we bred mice in which a floxed allele of the *Septin7* gene^17^ is recombined by Cre recombinase in Schwann cells under control of the *Cnp* promoter.^28^ Indeed, *Septin7*-cKO (conditional knock-out) mice lack both *Septin7* (Fig. 2D) and *Septin2* (Supplementary Fig. 4A) immunolabelling from Schwann cells, and thus presumably the entire myelin septin multimer.^14^ Here, patient serum reacted weakly with the axon while binding to paranodes, abaxonal myelin, and SLIs were lost in *Septin7*-cKO mice (Fig. 2D) as opposed to their flox/flox littermates (Supplementary Fig. 4B). Weak immunolabelling of SEPTIN7 in cKO axons was shown using the commercial SEPTIN7 antibody (Fig. 2D. In line with CNS pre-adsorption, IgG signals were lost on sciatic nerves upon pre-adsorption of sera with SEPTIN3, -5, -6, -7, -11 multimers Fig. 2E, serum insets). On a control human sural nerve biopsy, anti-SEPTIN7 staining confirmed the distribution of septin proteins in human nerves (Supplementary Fig. 4C).

### Septin multimer autoantibodies penetrate the node of Ranvier and SLIs in cultured dorsal root ganglion neurons

Nodo-paranodal structures and SLIs are hard to reach by autoantibodies.^29,30^ To assess binding to these structures in living and unfixed neurons, we performed pre-incubation assays on live murine myelinating DRG cell cultures.

**Figure 3:**
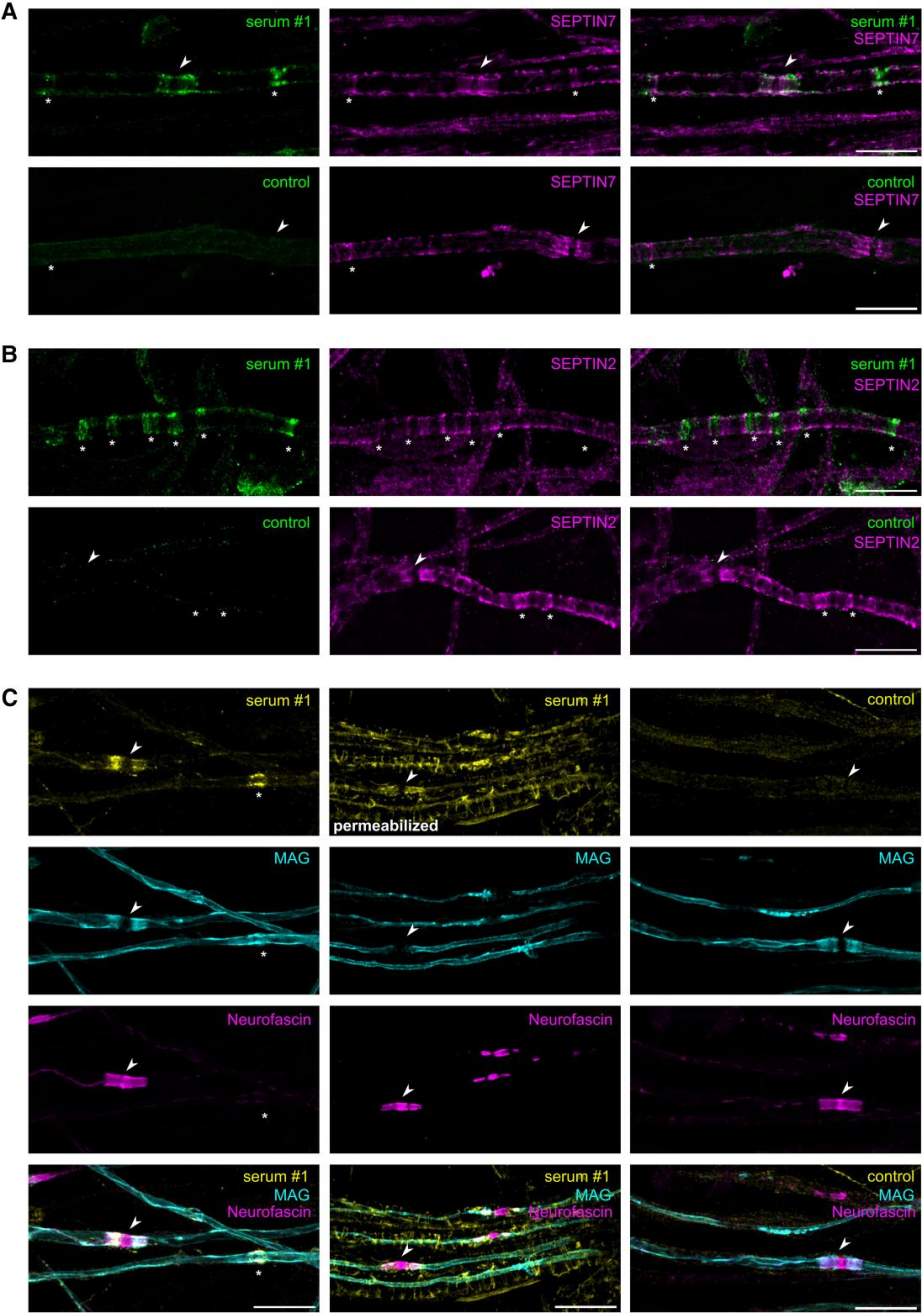
**Septin multimer IgG bound to paranodes and Schmidt–Lanterman incisures of living myelinated axons**. (**A** and **B**) Photomicrographs show serum binding of Patient 1 (green), but not of a healthy control serum to nodo-paranodal structures in living cultured myelinating dorsal root ganglion cultures after 5 days of preincubation. The patient serum’s binding partly co-localized with SEPTIN7 (**A**, magenta) and SEPTIN2 (**B**, magenta), which are expressed at the paranodes (arrowheads) and the Schmidt–Lanterman incisures (asterisk), as shown in the overlay image. Note that staining with commercial SEPTIN antibodies was performed after fixation and permeabilization while serum preincubation was done on live cells. Overlay of both signals is in particular visible at the SEPTIN7 positive paranode. (**C**) Despite strong binding of the patient serum to the paranodes after 5 days of preincubation (yellow, *left*), the compact myelin, nodo-paranodal region (arrowheads) and Schmidt–Lanterman incisures showed no morphological alterations, as shown by anti-myelin associated glycoprotein (MAG) staining (cyan) and anti-neurofascin staining (magenta). Nodo-paranodal morphology was unaltered compared with staining after permeabilization without preincubation (yellow, middle) or after 5 days of preincubation of a healthy control serum (yellow, *right*). Scale bars = 10 µm (**A**–**C**). All experiments were performed twice and no discrepancies in results were observed. IgG = immunoglobulin G.

On non-fixed, non-permeabilized myelinated cultures, no binding was observed after a short incubation of 1 h. After two incubation days, slight IgG deposition at the SLIs and paranodes was present with serum 1, but not with control serum, with increasing intensity after 5 days (Fig. 3A–C). Binding partially co-localized with SEPTIN7 and SEPTIN2, especially at the paranodal region (Fig. 3A and B). The staining with commercial anti-SEPTIN7 and SEPTIN2 was slightly weaker after preincubation with patient serum compared with the control serum, indicating either epitope masking or target antigen internalization (Fig. 3A and B). Strong binding of patient serum to paranodes and SLIs could also be detected when incubating after fixation and permeabilization of the cells (Fig. 3C). Non-specific binding to fibroblasts and cells in the culture was likewise observed in healthy controls (Supplementary Videos 1 and 2, available at Zenodo, doi:10.5281/zenodo.20059953 and doi:10.5281/zenodo.20101101). Alterations to nodo-paranodal structures, myelin and SLI could not be detected within the incubation of 5 days (see co-staining with antibodies against MAG and neurofascin; Fig. 3C and Supplementary Video 2). In septin multimer overexpressing HEK cells, however, no live-cell binding of serum was evident (Supplementary Fig. 6).

Hence, despite their intracellular location, septin multimer autoantibodies reach their target *in vitro* and predominantly bind to SLIs, where native cellular structure and epitopes are presumably preserved.

### Complement deposition and immune cell infiltration in sural nerve biopsy

**Figure 4:**
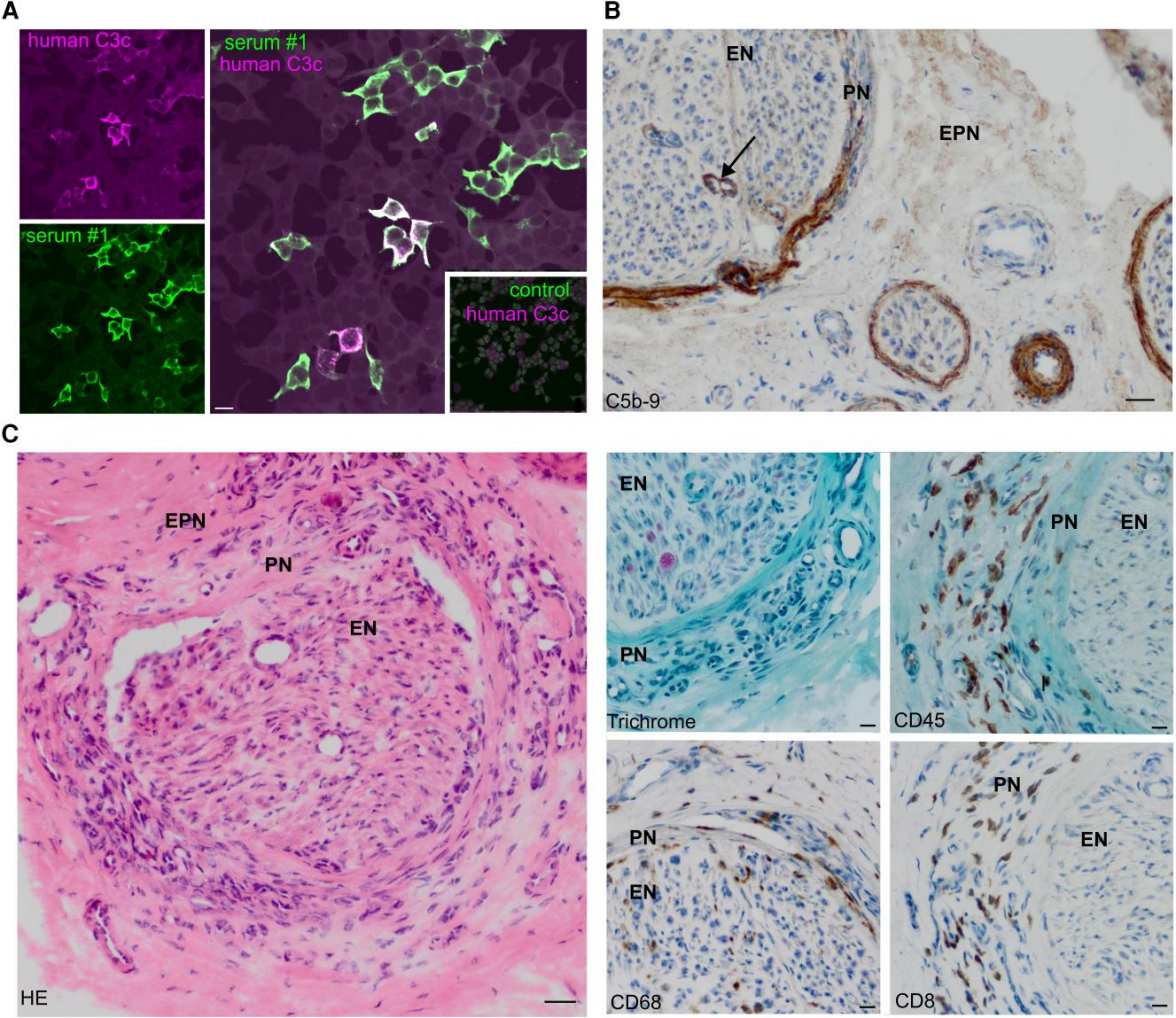
**Complement binding and sural nerve inflammation**. (**A**) Complement assay with septin multimer overexpressing HEK293 cells and control cells showed an overlay of Patient 1 serum immunoglobulin G (IgG) staining and commercial human C3c antibody while a control serum without septin multimer IgGs showed no complement deposition. Scale bar = 10 µm. (**B**) C5b-9 immunohistochemical preparation illustrating membrane attack complex deposition on an endoneurial (EN) capillary (arrow); note the physiological complement deposition in the perineurium (PN) and on epineurial (EPN) arterioles (original magnification ×200). (**C**) Haematoxylin and eosin (HE) as well as Gömöri trichrome stains of a sural nerve fascicle highlighting oedematous alterations and inflammatory infiltrates (CD68, CD45, CD8) predominantly in the perineurium (original magnification ×200). Scale bars = 50 µm (**B** and **C**).

IgG-subclass determination revealed a predominance of the complement fixing IgG3 and IgG1 subclasses (Patient 1: IgG3 > IgG1, Patient 2: IgG3 = IgG2 > IgG1, Patient 3: IgG1; data not shown). Further, we observed antigen-dependent complement deposition of cleaved complement component 3 (C3c) on fixed septin multimer overexpressing HEK cells upon incubation of serum 1 in contrast to the non-binding healthy control (Fig. 4A). In line, membrane attack complex deposition (illustrated via C5b-9) was visible in the sural nerve biopsy of Patient 1 located at endoneurial capillaries (Fig. 4B). Moreover, histopathological analysis of the sural nerve biopsy showed oedematous changes and inflammatory infiltrates, predominantly in the perineurium (Fig. 4C).

### Histopathological and ultrastructural *in vivo* evidence of axonal degeneration and de- and remyelination

**Figure 5:**
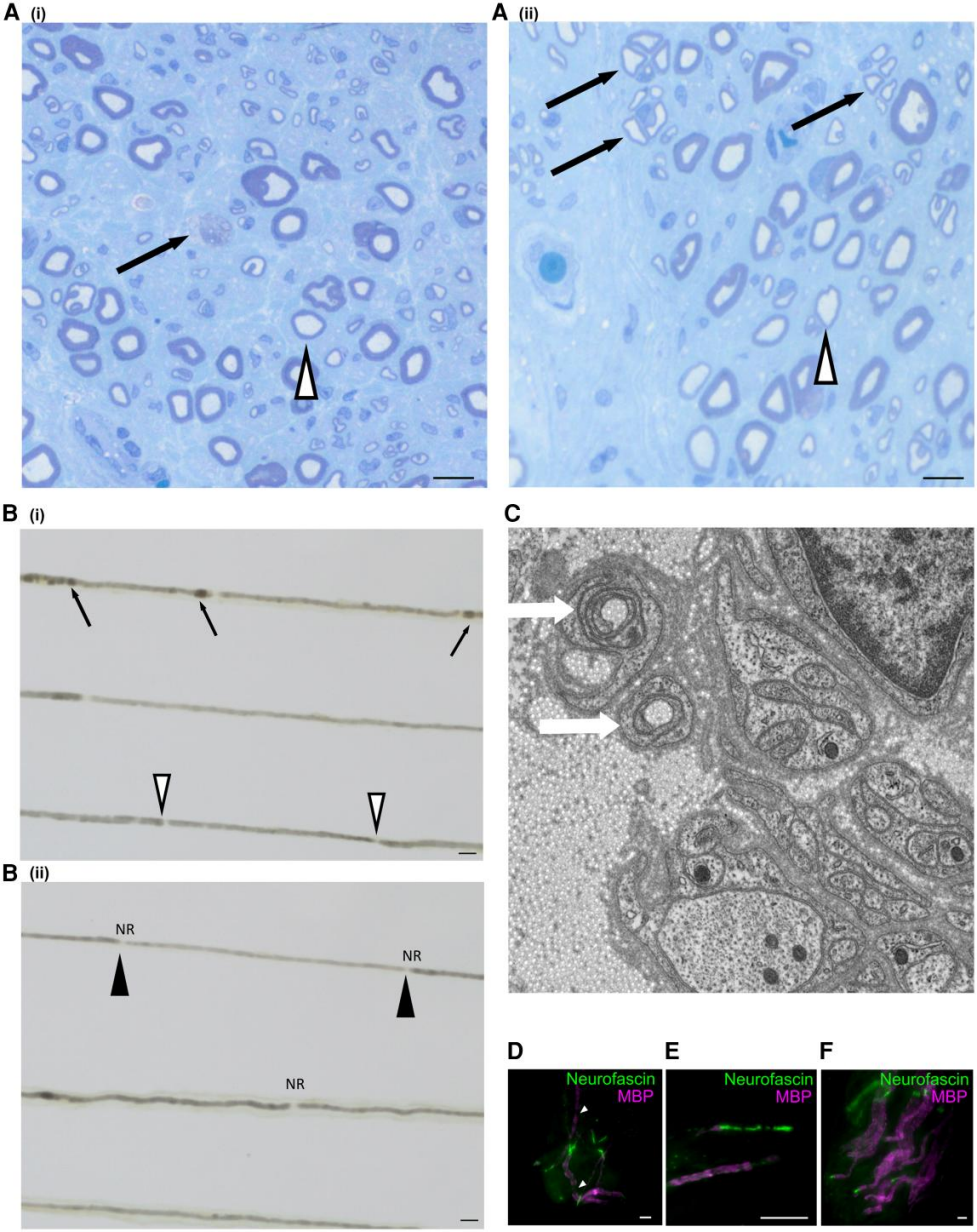
**Histopathological changes in myelin and axons in the sural nerve biopsy.** [**A**(**i** and **ii**)] Semithin sections stained by methylene blue illustrate acute axonal degeneration [e.g. filled arrow in **A**(**ii**)] as well as hypomyelinated fibres [arrowheads in **A**(**i** and **ii**)]. In addition, small clusters of axonal regeneration are also detectable [filled arrows in **A**(**ii**)] (original magnification ×600). [**B**(**i** and **ii**)] Fibre teasing shows myelin ovoids as signs of acute axonal degeneration [filled arrows in **B**(**i**)], short segments—in comparison to the one above, indicative of remyelination after primary demyelination [open arrowheads in **B**(**i**)]. Hypomyelinated shorter segments reflecting demyelination [filled arrowheads in **B**(**ii**)]. Nodal/paranodal regions did not show any overt enlargement of the paranodal area; NR = node of Ranvier (original magnification ×100). (**C**) Ultrastructural analysis revealed loss of unmyelinated axons, illustrated by the formation of collagen pockets (open arrows; original magnification ×12 000). (**D**–**F**) Representative images of indirect immunofluorescence on dermal punch biopsies of Patient 1 showed both reduced myelin staining (**D**, open arrowheads) and elongated nodes/paranodes demonstrated by elongation of anti-pan-neurofascin immunoreactivity (**E**), as well as regular nodal and myelin architecture (**F**). Scale bars = 50 µm (**A**–**D**); 10 µm (**F**–**H**).

Despite the absence of immediate structural effects by septin multimer autoantibodies on live DRG cultures, signs of severe de- and remyelination were evident in the sural nerve biopsy of Patient 1 with hypomyelinated axons and regenerating fibres, next to acute axonal degeneration [Fig. 5A(i and ii)]. Teased fibre preparation revealed acute axonal damage and some segmental demyelination, while nodal regions did not show distinct pathological changes, specifically no enlargement of the nodal gap [Fig. 5B(i and ii)]. Electron microscopy analysis showed loss of primary unmyelinated axons with the formation of collagen bundles surrounded by Schwann cell processes (Fig. 5C). In the skin, fibres showed partially decreased myelin staining (Fig. 5D), as well as elongated nodes of Ranvier in some fibres (Fig. 5E). However, normal fibres with regular myelin and intact nodal architecture were similarly present (Fig. 5F). Intraepidermal nerve fibre density was normal (data not shown). Taken together, morphological analysis of the sural nerve and the skin revealed myelin pathology next to axonal degeneration while the nodal regions were only partially abnormal.

### Clinical specificity of septin multimer antibodies

Accounting for the relevance of tissue reactivity, retrospective testing for septin multimer autoantibodies in different cohorts with motor-predominant neuropathies was performed using fixed CBAs, as well as brain and nerve tissue IIFAs. No additional case with septin multimer autoantibodies was detected in CIDP (*n* = 86), MMN (*n* = 18), GBS (*n* = 37) or ALS (*n* = 50). Additional neuropathy cohorts with diabetic neuropathies (*n* = 30), and other inflammatory neuropathies (*n* = 10) also remained without septin multimer autoantibody cases (Supplementary Fig. 5 and Supplementary Table 5). These findings indicate that septin multimer autoantibodies as detected by tissue IIFA and CBA are rare but specific.

### Immunotherapy likely stabilized disease progression

**Figure 6:**
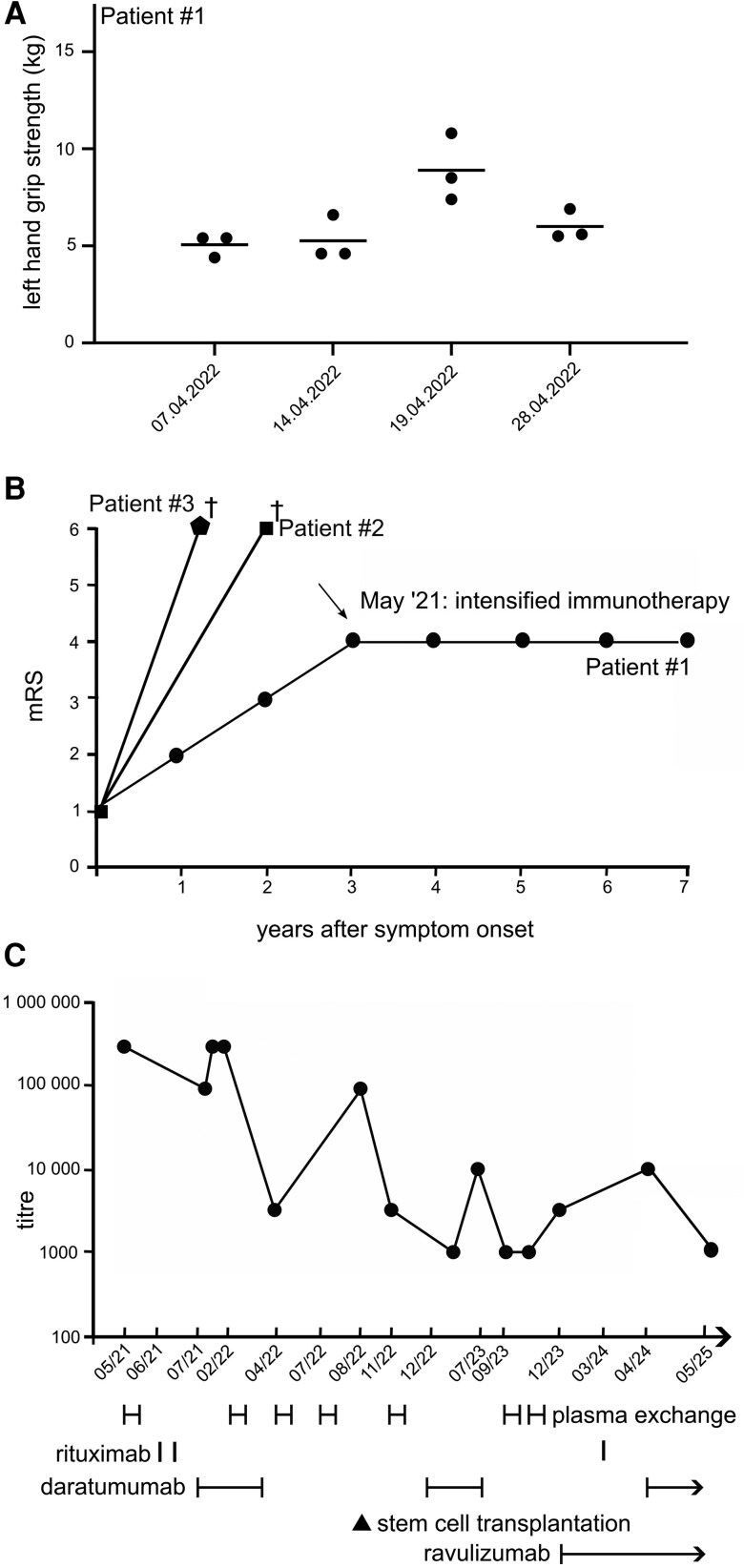
**Immunotherapy and cell-based assay titres in serum of Patient 1**. (**A**) Grip strength measurement (in kg) of the left hand before initiation of the second plasma exchange cycle (7 April 2022) until the day after the last plasma exchange treatment (28 April 2022) within that cycle. Dots represent three replicates of grip strength assessments per day and lines show the mean of the daily measurements indicating a slight improvement upon treatment. (**B**) Modified Rankin Scale (mRS) of Patients 1 (dots), 2 (rectangles) and 3 (polygon) at years after symptom onset. The arrow shows the initiation of intensified immunotherapy in Patient 1 in May 2021. Patients 2 and 3 died without effective immunotherapy (dagger) after 2 years and 14 months, respectively. (**C**) Septin multimer autoantibody titres in serum of Patient 1 from initiation of intensified immunotherapy onwards. Titres are displayed on a log10 scale. Black dots represent titres.

Based on these findings, we initiated extensive immunotherapy in Patient 1, including plasma exchange, B-cell and plasma cell depletion (rituximab, daratumumab), autologous stem cell transplantation and complement inhibition (ravulizumab). Plasma exchange was followed by immediate improvement in hand muscle strength (Fig. 6A) and a subjective reduction in sensory deficits. With intensified treatment, his overall condition stabilized at a low functional level (modified Rankin Scale = 4), corresponding with a decline in CBA titres (Fig. 6B and C). However, slow progression of muscle weakness over subsequent years could not be fully halted (data not shown), parallel to continuous autoantibody presence in serum (Fig. 6C). Patient 2 died 2 years after symptom onset, before the discovery of the septin multimer autoantibodies (Fig. 6B). Patient 3 received a trial of IVIg, and a single dose of rituximab based on a low-positive VGCC-P/Q antibody result (0.05 nmol/l; reference <0.02 nmol/l), but without any apparent clinical benefit (data not shown). The patient ultimately opted for hospice care before the septin multimer autoantibody was identified and died 14 months after disease onset.

## Discussion

We identified autoantibodies targeting septin multimers in peripheral nerve myelin. Three patients harbouring these autoantibodies were diagnosed with the LMND variant of ALS. While two patients did not receive effective intensified immunotherapy and died from rapidly progressing disease, one patient was extensively treated followed by stabilization of disease for several years. Experimental data showed that these autoantibodies reached SLIs and paranodes *in vitro* and initiated complement deposition, compatible with a potential pathogenic role. *In vivo* data from the sural nerve and skin biopsies revealed inflammation as well as myelin and axonal pathology.

The concept of autoreactive antibodies targeting the node of Ranvier is well-established^31,32^ and autoantibody-targeted therapy with plasma exchange and rituximab in autoimmune nodopathies has shown beneficial effects.^32^ The autoantibodies identified in this work, however, differ in several aspects from the autoantibodies targeting, for example, pan-neurofascin or contactin-1. While these antibodies bind the node,^21,33^ are often of IgG4-subclass,^34^ target extracellularly located proteins, and lead to immediate and direct pathological changes in (para)nodal ultrastructure,^21,29^ autoantibodies targeting intracellularly located septin multimers bound primarily to SLIs and paranodes, were predominantly of IgG3- and IgG1-subclasses, and showed no immediate effect in cultured myelinating DRGs. In line, the clinical presentation of all three patients suggests a chronic progressive process without definitive improvement upon treatment but rather disease stabilization, whereas autoimmune nodopathies usually present as (sub)acute demyelinating inflammatory neuropathies with good immediate response to appropriate immunotherapy.^31,35^

Septin multimers represent intracellular antigens; however, both our work and a previous study^6^ demonstrate their accessibility in live-cell systems. The work by Hinson *et al*.^6^ showed live-cell binding to primary hippocampal neuronal cultures and demonstrated functional effects for septin autoantibodies targeting neuronally expressed septin monomers. We also observed neuronal binding in live-cell neuronal cultures (Supplementary Fig. 6) supporting the accessibility of septin multimers in neuronal and myelinating cells. In contrast, no live-cell binding was observed in an artificial HEK293 cell overexpression system, suggesting that extracellular accessible epitopes might only be presented in the native cellular context.

Previous studies demonstrate that myositis-specific autoantibodies, including anti-signal recognition particle (SRP) and anti-3-hydroxy-3-methylglutaryl-coenzyme A reductase (HMGCR), can be pathogenic by not only inducing muscle damage *in vivo* through passive transfer but also by being internalized into muscle fibres where they disrupt intracellular targets, establishing a causal role for autoantibodies against intracellular antigens in myositis pathogenesis.^36,37^ These findings support the broader concept that antibodies against non-cell-surface proteins can exert pathogenic effects. However, direct proof of autoantibody-mediated pathophysiology of the here-described autoantibodies is still lacking. Even in the absence of direct pathogenicity, autoantibodies against intracellular antigens have been established as robust biomarkers in several autoimmune diseases. Accordingly, the absence of septin multimer autoantibodies in multiple disease cohorts supports their potential utility as a disease-specific biomarker.

One of the predominant binding sites of the autoantibodies described here within the PNS is the SLI. Further, the available nerve biopsy revealed distinct immune-mediated abnormalities including features of de- and remyelination but also extensive axonal damage. These findings raise the possibility that a long-term demyelinating and subsequent early axonal degeneration process could be initiated at and spread from the SLIs. Septin multimer autoantibodies may thus define a novel subgroup of autoantibody-associated neuropathies, which could be termed ‘incisuropathies’, characterized by impaired metabolic exchange and/or disruption of myelin structural integrity potentially leading to early axonal irreversible damage.

The direct effect of septin deficiency or overexpression in PNS myelin is largely unexplored. Septin abundances are altered in genetic demyelinating neuropathy mouse models^38,39^ and *Septin9* mutations cause hereditary neuralgic amyotrophy,^40,41^ often associated with prominent axonal damage in electrophysiology.^42^ However, mice lacking *Septin2* or *Septin9* specifically in Schwann cells did not show an evident neuropathic phenotype.^14^ Long-term effects (>1 year) such as maintenance of myelin integrity were, however, not investigated in *Septin2*-cKO or *Septin9*-cKO mice,^14^ nor in our cultured DRGs upon autoantibody incubation. Yet a potential function of septins on PNS myelin maintenance (rather than development) appears possible when considering their function in the maintenance of the CNS myelin structure.^43^

Complement plays a key role in several autoantibody-mediated neuropathies, including myasthenia gravis, where its inhibition is effective in treatment.^44,45^ Septin multimer autoantibodies were mainly IgG3, a complement-activating subclass previously linked to severe autoimmune nodopathies with pan-neurofascin and contactin-1 IgG3 and fulminant course.^46,47^ The nerve biopsy showed inflammation including endoneurial capillary complement deposition. Although overlapping with further immunotherapies, Patient 1 received complement inhibitors (ravulizumab), and intermittent clinical stabilization might have suggested a contribution to disease pathology.^46,47^

However, extensive treatment in one patient including complement inhibition, could not reverse the clinical course, consistent with early axonal damage, advanced atrophic paresis and persistent high autoantibody titres. Earlier autoantibody testing and aggressive intervention might have prevented irreversible damage, underscoring the value of early biomarker screening.

### Limitations

Septin multimer autoantibodies seem to be rare as only three cases were identified within different neuroimmunology laboratories over several years, thus limiting the generalizability of our findings given the small number of patients reported. Neuropathic features in large motor fibres could not be assessed, and direct autoantibody pathogenicity remains unclear, as no motor nerve cell culture model or animal testing was conducted. The complement deposition assay was performed on fixed cells overexpressing septin multimers as cell surface expression could not be achieved in this system, thereby limiting the interpretation regarding potential complement activation *in vivo*. Further, IP-MS, HEK live-cell, DRG and complement studies were restricted to one serum sample due to limited availability of serums 2 and 3.

## Conclusion

Septin multimer autoantibodies may occur in motor-predominant and severe neuropathies. Immunotherapy should be considered if signs of inflammatory demyelination are seen in electrophysiology or histology. Future studies should clarify their frequency and relevance as biomarkers and assess pathogenicity using patient-derived monoclonal antibodies or active immunization in animal models, as well as therapy responses in larger cohorts.

## Supplementary Material

awag183_Supplementary_Data

## Data Availability

Data are available from the principal investigator upon reasonable request.
