# Supplementary material for: Septin multimer autoantibodies in severe motor neuropathy mimicking lower motor neuron disease
Source: Brain. 2026 Jun 8;149(8):2731–46. doi: 10.1093/brain/awag183 (PMC13431799; doi:10.1093/brain/awag183)
Supplement: awag183_Supplementary_Data [file awag183_supplementary_data.zip › brain-2025-02186-File010.pdf]

Supplementary Material

Arlt, Miske, Appeltshauser et al.

Supplementary Methods

1. Indirect immunofluorescence assay (IIFA) screening on brain tissue

1.1. Charité protocol – research-based tissue IIFA

Unfixed full brain slides of mice were were blocked with blocking solution (10 % normal goat serum, 2.5 % bovine serum albumin) for 1h at room temperature (RT). Sera (screening dilution 1:200 in blocking solution) were added over night and kept at 4°C. After 3x5min washes with PBS, secondary antibodies labelled with Alexa-488 against human IgG (#109–545-003, Dianova, Hamburg, Germany, 1:1,000) were added for 2h at RT and protected from light. After additional 3x5min washes with PBS, slides were mounted and kept at 4°C.

1.2. Mayo Clinic protocol – clinically validated tissue IIFA

Brain tissue composites of mice were post-fixed with 4% PFA for 1min at RT and permebealized with 0.5% 3-[(3-cholamidopropyl)dimethylammonio]-1-propanesulfonate (CHAPS) for 1min at RT, washed with ice-cold PBS, and blocked with 10% normal goat serum (NGS) in PBS for 1h at RT. Meanwhile, sera were preabsorbed with bovine liver powder for 1h at RT to absorb non-specific autoantibodies, and centrifuged for 10min at 14.000 rpm. The supernatant with a final serum screening dilution of 1:240 was added onto the post-fixed and permebealized brain composite slides for 1h at RT. After washing with PBS 3x5min, secondary antibodies directed against human immunoglobulin G (IgGs) labelled with FITC (#2040-02 Southern biotech, AL, USA, 1:200) were added for 1h at RT and slides were protected from light. Slides were mounted after washing with PBS.

1.3. EUROIMMUN protocol – clinically validated tissue IIFA

Sera were diluted in PBS using defined screening dilutions starting at 1:10 up to 1:100. PBS-diluted sera were added to unfixed cryosections of rat hippocampus and cerebellum for 30min at RT, rinsed with PBS-Tween and then washed in PBS-Tween for 5min. . Secondary Alexa488-labeled antibodies against human IgGs (Jackson ImmunoResearch, Suffolk, United Kingdom, 1:500)) were added for 30min at RT and unbound antibodies were washed off with PBS-Tween. If required, cell nuclei were stained with TO-PRO-3 iodide (Thermofisher Scientific, final dilution of 1:2,000) prior to mounting.

2. Production of recombinant SEPTIN2,6,7,9 multimers

Recombinant SEPT2/6/7/9 complexes were purified from bacteria. E. coli BL21(DE3) strain bacteria were transformed with plasmids encoding SEPT2/6 (pnEA-vH_His-TEV-SEPT2_SEPT6; gift from Monos Mavrakis, addgene plasmid #174497; http://n2t.net/addgene:174497; RRID: Addgene_174497[1])) and SEPT7/9 (pnCS_SEPT7_SEPT9_i1-TEV-Strep; gift from Manos Mavrakis, addgene plasmid # 174500; http://n2t.net/addgene:174500; RRID: Addgene_174500[1]). After selection with ampicillin and spectinomycin, a single colony was picked and inoculated in LB with the same antibiotics, and cultured at 37°C until they reached an OD of 0.6-0.8. Following induction with 0.5 mM IPTG for 16h at 18°C, bacteria were pelleted and resuspended in lysis buffer containing 100mM Tris-HCl pH 8, 150mM NaCl, 1mM EDTA, 1mM DTT, 1mM PMSF and 1mg/ml DNase, and sonicated. Lysates were cleared of debris by centrifugation at 17,000 RPM and the supernatant was filtered with a 0.45 micron pore syringe filter. Subsequently, SEPT2/6/7/9 complexes were isolated using sequential affinity and size exclusion chromatography with an AKTA pure FPLC system (Cytiva). The filtered lysate was injected through a 50ml Superloop (Cytiva 19785001) into an HisTrap HP 5ml column, which was equilibrated with a binding buffer containing 100mM Tris-HCl pH 8, 150mM NaCl, 1mM EDTA and 1mM DTT. After binding, the column was washed with wash buffer I (100mM Tris-HCl pH8, 150mM NaCl, 1mM EDTA, 1mM DTT and 15mM Imidazole pH 8) followed by a second wash with wash buffer II (100mM Tris-HCl, pH 8, 150mM NaCl, 1mM EDTA, 1mM DTT and 40mM Imidazole pH 8). The SEPT2/6/79 complex was eluted with buffer containing 100mM Tris-HCl pH 8, 150mM NaCl, 1mM EDTA, 1mM DTT and 250mM Imidazole pH 8) and collected in equal volume fractions. Fractions enriched with SEPT2/6/7/9 protein were pooled and injected into a StrepTrap XT 1 ml column (Cytiva 29401317), which was pre-equilibrated with the same binding buffer used with the HisTrap column. After sample application, the column was washed with five column volumes of binding buffer. SEPT2/6/7/9 complexes were eluted in two steps. In the first step, one column volume was eluted after applying an elution buffer containing 100mM Tris-HCl pH 8, 150mM NaCl, 1mM EDTA, 1mM DTT and 50mM biotin. Following this initial step, elution was paused for 30min, and resumed with four column volumes of the same buffer. Fractions that contained the SEPT2/6/7/9 complex were pooled based on protein absorbance at 280nM, and applied to a Superdex 200 Increase 10/300GL gel filtration column (Cytiva 28990944) - a size exclusion column for isolation of SEPT2/6/7/9 hetero-octameric complexes. Fractionation was performed with final storage buffer containing 50mM Tris-HCl pH 8, 300mM KCl, 5mM MgCl2 and 3mM DTT. Octamer identity and subunit composition was verified in purified fractions with SDS-PAGE and western blots.

3. Neutralization assays on tissue IIFA

Neutralization assays on tissue were performed as previously described.[2,3] Serum of patient 1 was incubated with different SEPTIN overexpressing HEK293 cell extracts (SEPTIN3,5,6,7,11 multimer,

SEPTIN3 alone, SEPTIN7 alone, a mixture of separately expressed SEPTIN3,5,6,7, and 11) or with extracts from empty-vector-transfecetd HEK293 cells as controls in a final dilution of 1:10 or 1:5 in PBS-Tween for 1h at RT. In case of preincubation with denatured SEPTIN3,5,6,7,11 multimers the extracts were incubated for 20min at 70°C before serum incubation. For pre-absorption with recombinantly made SEPTIN multimers (SEPTIN2,-6,-7 and -9), the serum of patient 1 was incubated with the septin multimer protein in a final dilution of 1:10 (serum 1:100, septin complex protein 1:10 (1mg/ml)) in PBS for 1h at RT. Pre-absorbed serum was subsequently added to brain and nerve tissue slides (hippocamus, cerebellum, and sciatic nerve teased fibers) for 30min at RT. After washing with PBS-Tween, slides were stained with secondary antibodies against human IgG labelled with FITC (EUROIMMUN) or Alexa-488 (Dianova or Jackson ImmunoResearch) for 30min at RT. For co-stainings with commercial SEPTIN antibodies illustrating the loss of binding upon SEPTIN HEK293 cell extract absorption, slides were subsequently stained with commercial anti-SEPTIN antibodies (SEPTIN2 - #ab179436, abcam, Cambridge, UK, 1:50 and SEPTIN7 - #18991, IBL-Tecan, Switzerland, 1:20) and secondary antibodies labelled with Alexa-594 (#111-585-003, JacksonImmunoResearch, Westgrove, Pennsylvania, U.S., 1:500). After washing with PBS-Tween, slides were mounted in mounting media.

4. Confocal colocalization studies on tissue IIFA

For colocalization studies, the sciatic nerve teased fiber IIFA protocol described in the main manuscript was used. After permebealization with 0.1% TritonX, blocking, serum, and anti-human IgG (Alexa-488) staining, commercial anti-SEPTIN antibodies against SEPTIN2 (abcam) and SEPTIN7 (IBL-Tecan) were diluted in PBS and incubated for 1h at RT. After washing, secondary anti-rabbit IgG antibodies labelled with Alexa-549 (JacksonImmunoResearch) were added for 1h at RT. Using this sequential approach, we minimized cross-reactivity of commercial SEPTIN antibody signals with human IgG antibody signals.

5. Live cell neuronal binding assay on primary hippocampal neurons

Live-cell neuronal binding assays on rat primary hippocampal neurons was done as previously described (Hinson et al 2022, Gilligan et al 2024). In brief, neurons were cultured from embryonic (E18-20) animals. Cells were plated in Neural Basal Plus media with B27 supplement (Gibco, Gaithersburg, MD, USA) in poly-L-lysine coated chamber slides (Corning) after Hanks Balanced Salt Solution (HBSS) incubation of the minced brain tissue. Seeding density was at $3x10^4$ cells/well. Medium exchange was perdormed every 4 days. At day 20-22 after plating, cells were subjected to live-cell staining with patient samples. CSFs (1:3 in PBS) and sera (1:20) were incubated for 30min on ice. After washing with ice-cold PBS, secondary antibodies against human IgG labeled with FITC (Southern biotech, 1:200 in PBS) were added on live-cells for 30min on ice. Cells were washed in PBS, and then

fixed in 4% PFA for 10min at RT, and subsequently permebealized with 0.2%TritonX for 3min at RT. After washing, cells were blocked with 10% NGS for 1h at RT, and commercial anti-SEPTIN antibodies (SEPTIN6 (#12805-AP, Proteintech) and SEPTIN11 (#14672-1-AP, Proteintech)) and an anti-TUBULIN antibody (anti-acetylated tubulin #T7451-100UL, ThermoFisher) were added over night at 4°C. After washing with PBS, secondary anti-rabbit antibodies labeled with TRITC (Southern biotech, 1:200), and anti-mouse antibodies labeled with Alexa-647 (#A21235, Invitrogen) were added for 1h at RT. Slides were washed in PBS and mounted in mounting media. Images were acquired on confocal microscopes. Fluorescent colours were retrospectively adjusted to be colour-blind safe.

6. Protein microarray

Protein array testing of serum was done as previously described[4,5]. In brief, sera of patient 1 and 3 were tested on the HuProt v4.0 protein microarray (CDI Laboratories, Puerto Rico) containing >21,000 GST-tagged recombinant human proteins pre-printed in duplicate on a nitrocellulose-coated glass microarray slide. Slides were thawed, blocked for 2h in 5% BSA/1× TBS-T, and incubated for 1h with serum (1:1,000). After washing, bound antibodies were detected with anti human IgG secondary antibodies labeled with Alexa-647 (1:1000; Southern Biotech) for 1h. Slides were washed, dried, and stored overnight at −20°C, followed by incubation with anti-GST IgG (1:1000; EMD Millipore) and a fluorescent anti-GST secondary antibody (Thermo Fisher Scientific), each for 1h. After washing and centrifugation, slides were scanned using a GenePix 4000B microarray scanner. Proteins were ranked by Alexa Fluor 647 signal intensity.

Supplementary Results

Identification of cases

The flowchart in Supplementary Figure 1 shows the algorithm of how the reported cases were identified. Among 727 consecutive samples tested on brain IIFA and nerve IIFA in Charité in 2021 (cohort 1), we identified the index case using immunoprecipitation coupled to mass-spectrometry (IP-MS) and subsequent CBA (patient 1) in collaboration with EUROIMMUN where the serum was sent for additional autoantibody testing. Next, we attempted to find additional cases using the characteristic staining pattern on brain IIFA (*staining the molecular layer of cerebellum and hippocampus, and a strong thalamus, usually exceeding the pansynaptic fluorescence in hippocampus, cerebellum, and cortex)* as this is routinely done in clinical service testing of the neuroimmunology laboratory in Mayo Clinic Rochester, which has one of the highest through-put numbers worldwide. In addition to the characteristic septin pattern on brain tissue IIFA, we additional excluded samples with septin monomer autoantibodies in CBA. Among two investigated cohorts, we identified two additional cases. Cohort 2 consisted of 2,809 samples collected over one year (09/2016-10/2017) and was previously published[6]. All samples of cohort 2 were tested for septin multimer autoantibodies at EUROIMMUN. Here, one additional case (one serum, no paired CSF available, patient 2) was identified as purely septin multimer reactive. Cohort 3 samples were preselected based on the above mentioned criteria: Out of 27 samples from consecutively tested samples in 2023 on IIFA at Mayo Clinic (total of 121,513 samples), seven had enough residual material remaining to be sent to EUROIMMUN for septin multimer autoantibody testing (cohort 3, n=7). From those seven cases, an additional two samples (one serum and CSF pair, patient 3; one CSF only) demonstrated septin multimer autoantibodies. The CSF only sample was extinguished and could not be tested on sciatic nerve teased fibers. Further, clinical data for that sample was unavailable.

The two additionally identified cases from cohort 2 and 3 with clinical data available also showed the characteristic novel pattern on sciatic nerve teased fibers, identitical to the one observed in the index patient 1. We retrospectively collected clinical and paraclinical data of those two patients in addition to the index patient #1.

Supplementary Figures

Supplementary Figure 1: Algorithm of case identification with septin multimer autoantibodies and lower motor neuron disease

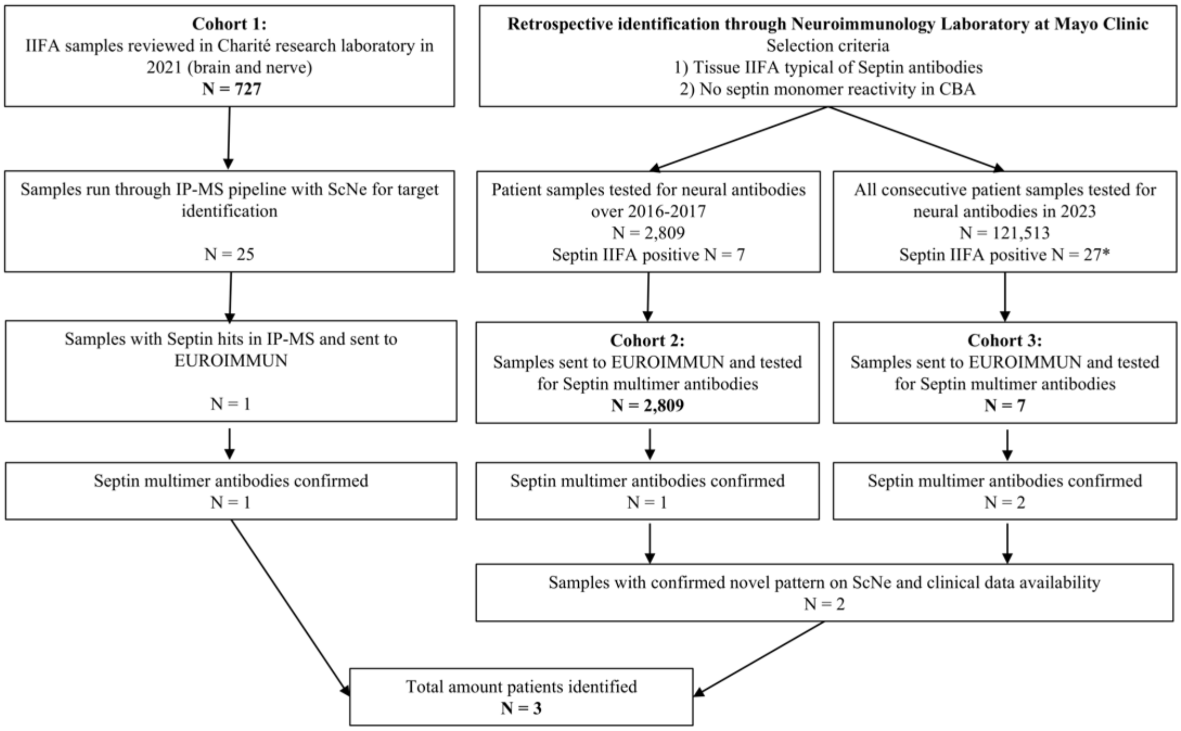

Supplementary Figure 1: IIFA: indirect immunofluorescence, IP-MS: Immunoprecipitation coupled to mass spectrometry, ScNe: sciatic nerve, CBA: cell-based assay. * N=7/27 samples with residual material were sent to EUROIMMUN for septin multimer antibody testing after pre-selecting samples according to IIFA criteria. Total amount of samples tested comprises cohort 1 (n=727) + cohort 2 (n=2,809) + cohort 3 (n=7), total n =3,543.

Supplementary Figure 2: Cell-based assays and protein microarray testing suggest a septin multimer epitope

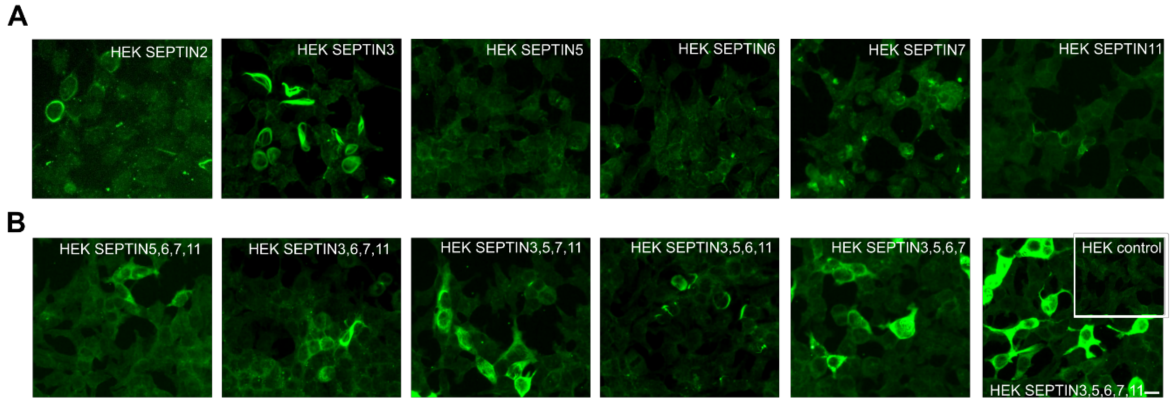

C

Patient	Protein	Rank
#1 serum	SEPT2	1,891
	SEPT2	3,170
	SEPT2	3,345
	SEPT9	3,349
	SEPT6	3,639
#3 serum	SEPT9	593
	SEPT2	730
	SEPT2	870
	SEPT9	2,303
	SEPT6	3,587

Supplementary Figure 2: (A-B) Representative images of patient 1 serum binding to HEK cells overexpressing septin monomers (2,3,5,6,7,11) (A) and septin multimers, each lacking a different monomer (B). Strong binding to the full complex composed of septin-3,-5,-6,-7,-11 as well as absence of binding to non-transfected cells are shown as controls at the end of panel B. C) Protein microarray analysis showing no relevant septin protein hits, indicating the absence of detectable linear epitope reactivity against septin proteins in patients 1 and 3. Serum from patient 2 was exhausted and unavailable for testing. On the protein microarray, proteins ranked among the top 50 are considered potential true autoantibody targets[4,5]. The highest-ranking SEPTIN protein was detected at position 593.

Supplementary Figure 3: Neutralization assays confirm a conformational septin multimer epitope

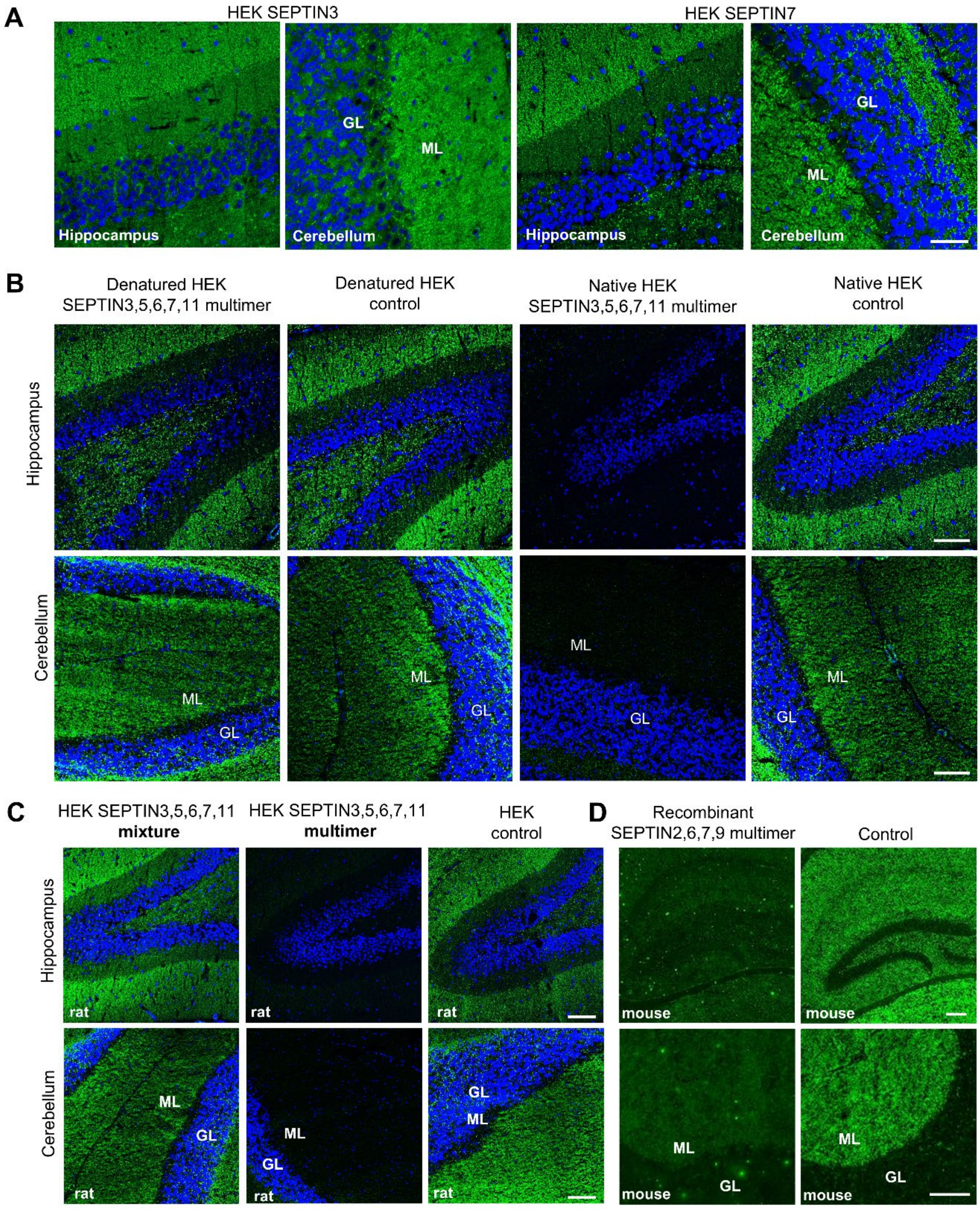

Supplementary Figure 3: (A) Neutralization assays using HEK cell extracts expressing SEPTIN3 and SEPTIN7 monomers only. Failure of IgG-preabsorption is demonstrated on unfixed brain tissue. (B) Neutralization assays using native versus heat-denatured HEK cell extracts expressing SEPTIN multimers and control HEK cells. To enable septin multimer formation, HEK cells were transfected with plasmids encoding for SEPTIN3, SEPTIN5, SEPTIN6, SEPTIN7, and SEPTIN11. Heat denaturation of extracts was performed at 70 °C for 20min. Native and denatured extracts from septin-

multimer-expressing HEK cells and control HEK cells were used to pre-absorb serum from patient 1 before serum incubation on unfixed rat brain tissue. Only the native septin multimer achieve efficient IgG-preabsoprtion suggesting epitope destruction with heat denaturation. (C) Neutralization assays using extracts from a mixture of HEK cells expressing individual septin monomers, septin-multimer-expressing cells, and control HEK cells. For monomer expression, HEK cells were transfected with a single plasmid encoding for either SEPTIN3, SEPTIN5, SEPTIN6, SEPTIN7, or SEPTIN11. To mimic the presence of multiple septin monomers, extracts from monomer-expressing cells were combined in equal proportions. These extracts, along with extracts from septin-multimer-expressing cells and control cells, were used to pre-absorb serum from patient 1 before serum incubation on unfixed rat brain tissue. The mix of septin monomer HEK cell extracts fails to pre-absorb the serum IgGs reinforcing true multimer-reactivity vs a combined reactivity to single septin monomers. Neutralization assays depicted in A-C were equally perfomed on monkey brain sections showing the same results (data not shown). (D) Neutralization assay using a recombinantly produced septin multimer (SEPTIN2, SEPTIN6, SEPTIN7, SEPTIN9) compared with a control condition lacking recombinant protein. The recombinant multimer protein was used to pre-absorb serum from patient 1 before serum incubation on PFA-fixed and CHAPS-permebealized mouse brain tissue. Pre-absoprtion is successful with the recombinant hetero-octamer composed of SEPTIN2,6,7,9 reinforcing the presence of a multimer-dependent epitope. Scale bar: 100µm.

Supplementary Figure 4: Septin multimer autoantibodies target primarily myelin-expressed septin multimers in the peripheral nervous system

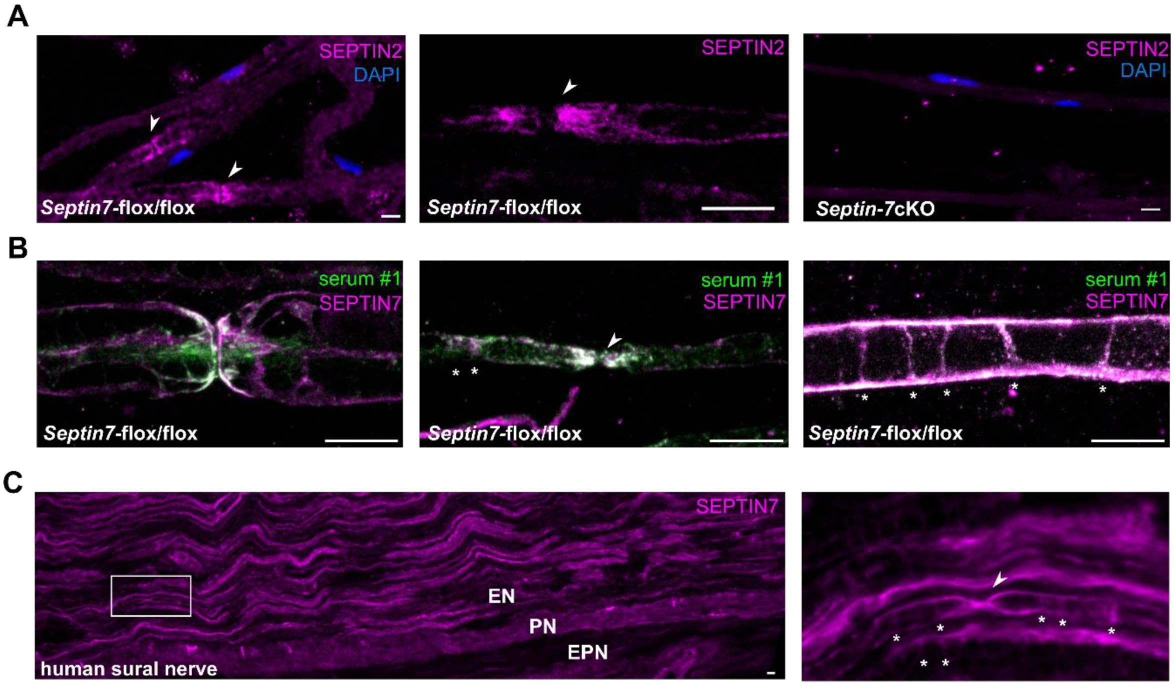

Supplementary Figure 4: A) SEPTIN2 commercial antibody reactivity (magenta) on *Septin7*-flox/flox mice and *Septin7* conditional knock-out (*Septin7*-cKO) littermates lacking *Septin7* expression in Schwann cells. On *Septin7*-flox/flox, SEPTIN2 expression is evident at the paranode while no reactivity is present on the *Septin7*-cKO nerves. Cell nuclei are counterstained with DAPI. B) Costaining of commercial SEPTIN2 and -7 antibodies with anti-septin multimer serum #1 on *Septin7*-flox/flox littermates of *Septin7*-cKO mice confirmed signal overlap at paranodal myelin, paranodes (arrowhead) and Schmidt-Lanterman incisures (SLIs) (asterisk). C) Longitudinal section of a control human sural nerve confirmed SEPTIN7 reactivity at paranodal myelin, and paranodes (arrowheads), as well as SLIs. EN: endoneurium, PN: perineurium, EPN: epineurium. Scale bar: 10µm.

Supplementary Figure 5: Peripheral neuropathy cohort screening on septin multimer autoantibody cell-based assay, rat brain, and mouse sciatic nerve teased fibers

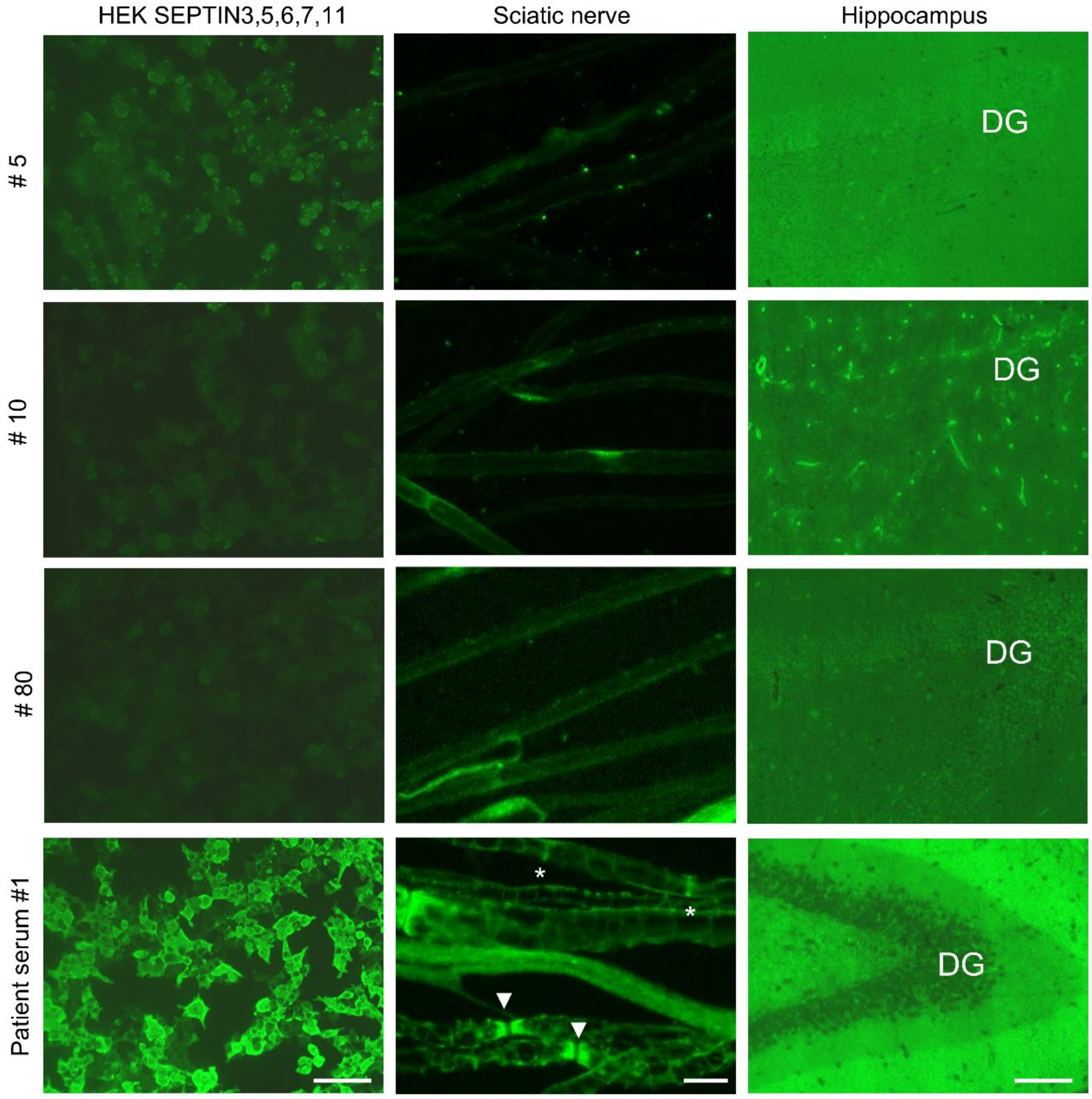

Supplementary Figure 5: Representative images of three different sera are shown demonstrating no specific SEPTIN IgG reactivity in cell-based assays (CBA) overexpressing septin multimers consisting of SEPTIN3,5,6,7,11, nor on tissue (rat brain and mouse sciatic nerve teased fibers). Diagnoses of sample #5 and #10: chronic demyelinating inflammatory polyradiculopathy, diagnosis of sample #80 : Guillain-Barré syndrome. Note the weak clumpy reactivity in disease cohort sample #5 on the septin multimer CBA in contrast to the homogenous staining of cells including their processes in the CBA of patient serum #1. Similar clumpy reactivities in CBA were observed in 13 / 213 cases. None of those samples showed SEPTIN IgG reactivity on brain (molecular layer staining) or nerve tissue (Schmidt-

Lanterman Incisures (stars) and paranodes (arrowheads). DG: Dentate gyrus. Scale bars in cell-based assay and hippocampus images: 100µm; scale bar in sciatic nerve teased fiber images: 10µm.

Supplementary Figure 6: Live-cell binding of septin multimer autoantibodies in HEK293 cells and hippocampal neurons

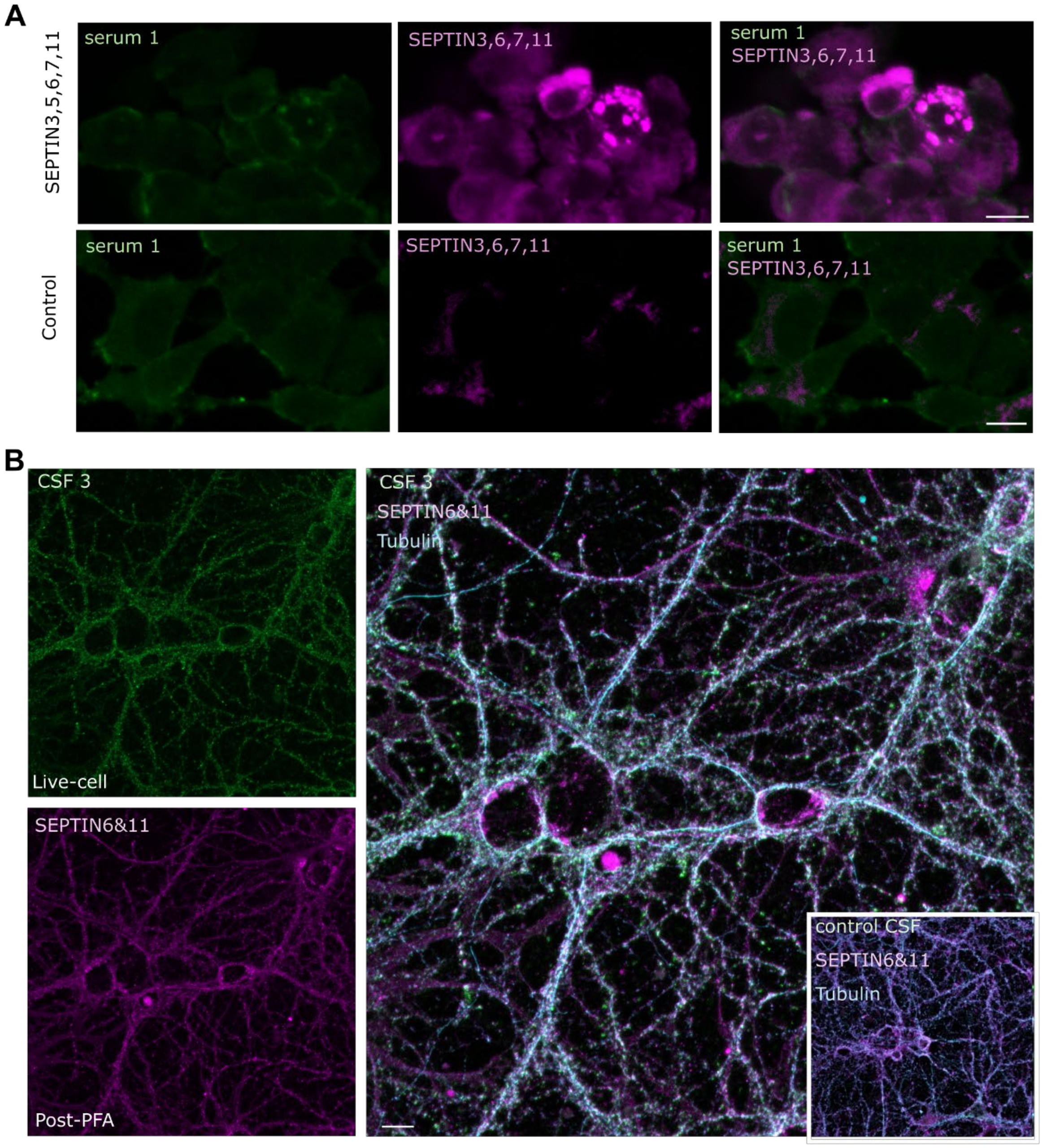

Supplementary Figure 6: (A) Representative images illustrating the live cell binding absence of serum #1 on septin multimer (SEPTIN3,5,6,7,11) overexpressing HEK cells (absence of green fluorescence, serum dilution 1:20) after 30min incubation on ice. In parallel a mix of commercial antibodies against septin monomers (SEPTIN3,6,7,11; dilution of 1:20) was tested and live-cell binding was equally not observed (data not shown). To illustrate intracellular septin expression, cells were fixed and permebealized with MeOH and stained with the commercial antibody mix (dilution 1:100) as represented by magenta (originally red) fluorescence in two adjacent cells. Colours of fluorescence were retrospectively adjusted to be colour-blind friendly. Control HEK cells show absence of live cell

binding and septin multimer expression. Live-cell binding on HEK cells was performed three times without discrepant results. (B) Representative images of live cell binding of the corresponding cerebrospinal fluid (CSF) of patient #3 on live cell hippocampal neuron cultures (DIV 22, final CSF dilution in PBS of 1:3) after 30min on ice. Post-fix counterstaining with commercial septin antibodies against SEPTIN6 and 11 (magenta) show partial overlay on the abaxonal membrane. Commercial septin antibody reactivity exceeds CSF live cell binding signal probably due to staining post-fixation and permebealization with PFA and TritonX. Post-fix tubulin staining (cyan) serves as a neuronal cell marker. Septin multimer reactivity in CSF was confirmed in septin multimer cell-based assays prior to this experiment (data not shown). Absence of live-cell binding of a control CSF without septin multimer IgGs is shown in the overlay image insert (absence of green fluorescence) while SEPTIN6 and 11 expression (magenta fluorescence) is evident. Scale bars: 10µm.

Supplementary Figure 7: VGCC-P/Q staining on sciatic nerve teased fibers

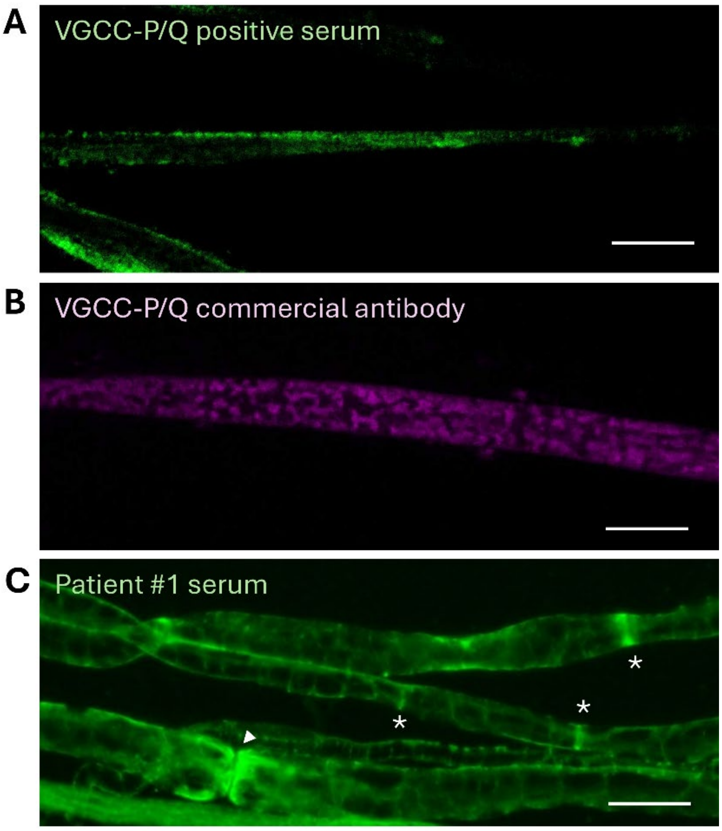

Supplementary Figure 7: A high titer positive sample for VGCC-P/Q antibodies (A) as detected by radioimmunoprecipitation assay (1.52nmol/l) was tested in parallel with a commercial antibody (B) (Atlas Antibodies, Stockholm, Sweden 1:50). Neither serum nor the commercial antibody demonstrated binding to Schmidt-Lanterman Incisures (stars) or paranodes (arrowhead), the novel binding pattern of septin multimer IgG (illustrated in C). VGCC: voltage-gated calcium channel complex. Scale bar:10µm.

Supplementary Videos

Supplementary Video 1: Serum IgG bound to paranodes and Schmidt-Lanterman incisures of living myelinated axons

The large-scale overview shows a triple-staining of a myelinated DRG, displaying the DRG explant on the right, and the fully myelinated axons with differentiated nodes of Ranvier and Schmidt-Lanterman incisures expressing MAG (cyan) on the left. The culture was incubated with serum of patient #1 before fixation and permeabilization. IgG deposits can be found at the nodes of Ranvier and the Schmidt-Lanterman incisures (yellow) and colocalize with SEPTIN7 (magenta). DOI: 10.5281/zenodo.20059953.

Supplementary Video 2: Morphology of the nodes of Ranvier remained intact after short-term serum preincubation

After short-term serum preincubation (patient #1, yellow) on living myelinated DRG neurons, the morphology of the nodes of Ranvier including nodal and paranodal region and the Schmidt-Lanterman incisures remains intact, as shown by MAG (cyan) and pan-Neurofascin (magenta) co-staining. DOI: 10.5281/zenodo.20101101.

Supplementary Tables

Supplementary Table 1: Electroneurographic findings in patient 1-3

Patient 1

A) Neurography 2018

Nerve	Distal motor latency (ms)	Amplitude dist. / prox. (uV/mV)	NCV (m/s)	F latency (ms)
Sural, left#	n/a	**--** (>3) ##	**--** (>41)	n/a
Tibial, right	6.33 (<6.5)	**1.27 / 0.92** (>3)	**26.0** (>40)##	**66.1** (<53)
Tibial, left	5.62 (<6.5)	3.5 / **2.3** (>3)**	**36.8** (>40)	**65.7** (<53)
Ulnar, motor right	3.39 (<3.5)	**6.0 / 5.0** (>7)	**41.4** (>53)	**32.3** (<30)
Ulnar, sensory right	n/a	12.1 (>10)	**38.9** (>45) ##	n/a
Median motor, right	**4.71** (<3.8)	**4.7 / 4.1** (>7)	**42.9** (>53)	**34.4** (<30)
Median, sensory right	n/a	35.5 (>10)	45.0 (>43)	n/a
Peroneal, right	**--**	**--**	**--**	n/a
Peroneal, left	**8.12** (<6.5)	**0.79 / 0.27** (>3) ###	**30.0 (>40)**	n/a

B) Neurography 2023

Nerve	Distal motor latency (ms)	Amplitude dist. / prox. (uV/mV)	NCV (m/s)	F latency (ms)
Sural, left#	n/a	**--** (>1,16) ##	**--** (>37.1)	n/a
Tibial motor, right#	**11.6** (<5.6) ##	**0.087** / **--** (>4.4)	**--** (>37.6)	n/a
Ulnar, motor right	3.56 (<3.6)	**1.67** / **1.51** (>7.3)	**48.4** (>48.7)	n/a
Ulnar, motor left	3.0 (<3.6)	**4.2** / **3.6** (>7.3)	**45.9** (>48.7)	**34.8** (<30)
Ulnar, sensory left	n/a	13.9 (>4.8)	50.3 (>47.6)	n/a
Median, motor left	**5.2** (<4.22)*	**0.28** / **0.23** (>4.7)	**36.7** (>49.1) *	**--** (<30)
Median, sensory left	n/a	27.4 (>6.6)	66.4 (>48.4)	n/a
Median, motor right	**4.9** (<4.2)	**0.93** / **0.89** (>4.7)	**46** (>49.1)	**--** (<30)
Median, sensory right	n/a	30.3 (>6.6)	62.0 (>48.4)	n/a

--: not reproducible, n/a: not applicable, NCV: nerve conduction velocity. Units and normal reference values are listed in brackets. Pathological values are highlighted in bold writing. ##Values in line with EAN/PNS CIDP criteria. Note the change of reference values in 2020. Stimulation was supramaximal on all stimulation sites except for **left tibial nerve. #Reduced reproducibility due to leg edema. *interpreted as carpal tunnel syndrome. ###technically, values in line with EAN/PNS CIDP criteria, but clinically not rated as conduction block due to dispersion and low overall amplitudes. In conclusion, EAN/PNS electrodiagnostic criteria meet the definition of “possible multifocal CIDP”. Clinical EAN/PNS criteria for “multifocal CIDP” are not met (no supportive criteria fulfilled, more probable alternative diagnosis).

Patient 2

A) Neurography 2017

Nerve	Distal motor latency (ms)	Amplitude dist. / prox. (uV/mV)	NCV (m/s)	F latency (ms)
Sural, left	n/a	8 (>0)	n.d.	
Ulnar, motor left	2.6 (<3.6)	6.2 (> 6.0)	64 (>51)	
Ulnar, sensory left	n/a	15.0 (>10.0)	60 (>54)	
Median motor, left	3.8 (<4.5)	**1.4** (>4.0)	59 (>48)	
Median, sensory left	n/a	**13.0** (>15.0)	57 (>56)	
Median motor, right*	**5.3** (<4.5)*	**1.0** (>4.0)	53 (>48)	
Median sensory, left*	n/a	**9.0** (>15.0)*	n.d.	
Peroneal, left	4.5 (< 6.6)	3.0 (>2.0)	49 (<41)	54.5 (<58)

NCV: nerve conduction velocity. Units and normal reference values are listed in brackets. Pathological values are highlighted in bold writing.. Stimulation was supramaximal on all stimulation sites (distal stimulation reported). *interpreted as carpal tunnel syndrome. Clinical EAN/PNS criteria for "multifocal CIDP" are not met (no supportive criteria fulfilled, more probable alternative diagnosis).

<u>Patient 3</u>

A) Neurography 2023

Nerve	Distal motor latency (ms)	Amplitude dist. / prox. (uV/mV)	NCV (m/s)	F latency (ms)
Sural, left	n/a	**--** (>6.0) ##	**--** (>40)	n/a
Sural, right	n/a	**--** (>6.0) ##	**--** (>40)	n/a
Peroneal, left	**--** (< 6.8)	**--** (>5.1)	**--** (>43)	n/a
Peroneal, right	**7.2** (< 6.8)	**1.3 / 1.1** (>5.1)	47 (>43)	n/a
Tibial, left	**10.3** (< 6.1) ##	**2.3 / 0.6****	43	n/a
Tibial, right	**9.8** (< 6.1) ##	**2.1 / 0.3****	41	n/a
Median, motor, left	**4.81** (<4.5)	**1.4 / 1.3** (>4.0)	50 (> 48)	n/a
Median sensory, left	n/a	15 (> 15.0)	**40** (>56)##	n/a
Ulnar, motor, left	2.54 (<3.6)	**2.7 / 2.3 / 2.3** (>6.0)	53 (>51)	n/a

NCV: nerve conduction velocity. n/a: not assessed /applicable. Units and normal reference values are listed in brackets. Pathological values are highlighted in bold writing. ##Values in line with EAN/PNS CIDP criteria. Stimulation was supramaximal on all stimulation sites except for **tibial nerve. In conclusion, EAN/PNS electrodiagnostic criteria meet the definition of “multifocal CIDP”. Clinical EAN/PNS criteria for “multifocal CIDP” are not met (no absent tendon reflexes in affected limbs, more probable alternative diagnosis).

Supplementary Table 2: Electromyography findings in patient 1 & 2

<u>Patient 1</u>

Muscle	interpretation	Spontaneous activity (SA)			Voluntary activity (VA)						
		**fib**	**psw**	**fasc**	**crd**	**amp**	**dur**	**poly**	**ip**	**recruit**	**MUP Amplitude**
Masseter	Normal	0/10	0/10		0					**late**	No analysis
Paravert.	**Moderately pathologic SA, slightly altered VA**	**2/10**	**1/10**		0	+	n	n	n	n	0,3-**4mV**
Deltoid	**Moderately pathologic SA and VA**	**3/10**	**2/10**	+	++	+	n	n	**-**	n	0,4-**3,9mV**
EDC	**Severely pathologic SA and VA**	**6/10**	**4/10**	++	+	+++	++	+	**-**	**late**	0,2-**9,5mV**
RF	**Severely pathologic SA and VA**	**5/10**	**3/10**	++	+	+++	++	n	**--**	n	0,5-**12mV**
TA	**Severely pathologic SA, no VA**	**6/10**	**4/10**	+	++				**--**		**No VA**
IOD I	**Severely pathologic SA, slightly pathologic VA**	**4/10**	**2/10**	+	++ +				**--**	n	0,2 – 0,37mV

The table shows electromyography results assessed between 2018 – 2023, with the worst test result reported. Pathological values are marked with bold letters. All muscles measured on the right side, EDC: Extensor Digitorum communis; N: normal, fib: fibrillations, psw: positive sharp waves, fasc: fasciculations, crd: complex repetitive discharges, amp: amplitude or motor unit potentials, dur: duration of motor unit potentials, poly: polyphasia, IOD = Interosseus dorsalis, ip: interference pattern, recruit: recruitment pattern, MUP: motor unit potential amplitude, RF: Rectus femoris, SA: spontaneous activity, TA: Tibialis anterior, VA: voluntary activity, + slightly pathologic /present, ++ moderately pathologic /present, +++ highly pathologic / present. – reduced, -- strongly reduced.

Patient 2

Muscle	interpretation	Spontaneous activity (SA)			Voluntary activity (VA)						MUP Amplitude
		**fib**	**psw**	**fasc**	**crd**	**amp**	**dur**	**poly**	**ip**	**recruit**	
Masseter	Normal	-	-		-						n/a
Paravert.	**Moderately pathologic SA**	+	n/a		n/a	+	n	n	n	n	n/a
APB	**Severely pathologic SA and VA**	+++	n/a	+	n/a	++	++	n/a	**-**	**late**	n/a
IOD I	**Severely pathologic SA and VA**	++	n/a	**-**	n/a	++	++	n/a	**-**	n/a	n/a
BB	**Moderately pathologic SA and VA**	+	n/a	++	n/a	+	++	n/a	**-**	**late**	n/a
RF	**Moderately pathologic SA, normal VA**	-	n/a	+	n/a	n	n	n/a	n	n/a	n
TA	**Moderately pathologic SA and VA**	+	n/a	+	n/a	+	+	n/a	**-**	n/a	n/a

The table shows electromyography results assessed between 2018 – 2023, with the worst test result reported. Pathological values are marked with bold letters. All muscles measured on the right side, APB: Abductor pollicis brevis, BB: Biceps brachii, fib: fibrillations, psw: positive sharp waves, fasc: fasciculations, crd: complex repetitive discharges, amp: amplitude or motor unit potentials, dur: duration of motor unit potentials, poly: polyphasia, IOD: Interosseus dorsalis, ip: interference pattern, recruit: recruitment pattern, MUP: motor unit potential amplitude, n: normal, n/a: not assessed, RF: Rectus femoris, SA: spontaneous activity, TA: Tibialis anterior, VA: voluntary activity, + slightly pathologic /present, ++ moderately pathologic /present, +++ highly pathologic / present. – reduced, -- strongly reduced.

Patient 3 showed normal values in all tested mucles, not shown. APB which clinically showed atrophy was not tested.

Supplementary Table 3: Motor Evoked Potentials in patient 1

**A) 2018**	**Arms**		**Legs**	
	Right	Left	Right	Left
Cercival/ lumbar latency (ms)	14.9	15.3	17.3	17.0
total latency (ms)	23.4	22.8	31.4	32.9
“central” motor latency (Δ total –lumbar/cervical)	8.5	7.5	14.1	15.9
Amplitude (cortical, mV)	0.57	0.17	0.16	0.31
Amplitude (cervical / lumbar, mV)	6.5	2.3	0.32	0.92
Interpretation	Normal	Normal	Normal	Normal

B) 2023

	**Arms**		**Legs**	
	Right	Left	Right	Left
Cercival/ lumbar latency (ms)	17.4	17.6	n/a	n/a
total latency (ms)	25.0	24.5	n/a	n/a
“central” motor latency (Δ total –lumbar/cervical)	7.4	7.1	n/a	n/a
Amplitude (cortical, mV)	0.41	0.19	n/a	n/a
Amplitude (cervical / lumbar, mV)	0.17	0.1	n/a	n/a
Interpretation	Normal “central” prolonged total latency	Normal “central” prolonged total latency	Not measurable	Not measurable

Supplementary Table 4: Autoantibody panel evaluation in serum

Number	Positive result	Negative result
Patient 1	Positive indirect immunofluorescence on rat brain* (1:3200)	ANNA-1, ANNA-2, ANNA-3, Yo, Tr/DNER, Ma, GAD65, Amphyphysin, Aquaporin-4, MOG, NMDA-R, AMPA-R, GABA-B-R, LGI1, CASPR2, IgLON5, DPPX, CARPVIII, Glycin-R, mGluR1, mGluR5, GABA-A-R, Rho GTPase activating protein 26, Recoverin, GluRD2, Flotillin, ITPR1, Homer3, Neurochondrin, Neurexin-3-alpha, ERC1, Sez6l2, AP3B2, Contactin1, Neurofascin155, Neurofascin186, APT1A3, KCNA2, Dopamin-R
Patient 2	Calcium Channel Ab P/Q type 0.05 nmol/L (Ref: <0.02 nmol/L)<br>Positive indirect immunofluorescence on mouse brain* (1:480)	Amphyphysin Ab, AGNA-1, ANNA-1, ANNA-2, ANNA-3, CRMP-5-IgG, PCA-Te, PCA-1, PCA-2),AMPA-R, CASPR2, GABA-B-R, LGI1, NMDA-R, Ach-R, VGCC-N-Type, AchR Ganglionic Neuronal, VGKC, GAD65, IgG GM1, IgM GM1, IgG GD1b, IgM GD1b
Patient 3	Calcium Channel Ab P/Q type 0.05 nmol/L (Ref: <0.02 nmol/L)<br>Positive indirect immunofluorescence on mouse brain* (1:3840)	Amphyphysin, AGNA-1, ANNA-1, ANNA-2, ANNA-3, CRMP-5-IgG, PCA-Te, PCA-1, PCA-2, AMPA-R, CASPR2, DPPX, GABA-B-R, LGI1, NMDAR-R, AP3B2, mGluR1, GRAF1, ITPR1, Neurochondrin, SEPTIN5, SEPTIN7, VGCC-N-Type, VGKC

* Endpoint titer in parenthesis.

Supplementary Table 5: Retrospective screening in disease cohorts for septin multimer autoantibodies

Cohort	Number	Assays tested	Result
Chronic inflammatory demyelinating polyradiculopathy	86	CBA, brain IIFA, nerve IIFA	No positive case
Guillain-Barré syndrome	37	CBA, brain IIFA, nerve IIFA	No positive case
Multifocal motor neuropathy	18	CBA, brain IIFA, nerve IIFA	No positive case
Amyotrophic lateral sclerosis	50	CBA, brain IIFA, nerve IIFA	No positive case
Diabetic neuropathy	30	CBA, brain IIFA, nerve IIFA	No positive case
Anti-MAG neuropathy	4	CBA, brain IIFA, nerve IIFA	No positive case
Chronic inflammatory demyelinating polyradiculopathy variants	3	CBA, brain IIFA, nerve IIFA	No positive case
Paraproteinaemic neuropathy	1	CBA, brain IIFA, nerve IIFA	No positive case
Anti-NF155 nodopathy	1	CBA, brain IIFA, nerve IIFA	No positive case
Paraneoplastic neuropathy	1	CBA, brain IIFA, nerve IIFA	No positive case
Multiple Sclerosis	50	CBA only	No positive case

Retrospective testing in different neuropathy cohorts as well as amyotrophic lateral sclerosis, and multiple sclerosis were performed. Among all cases tested on all assays, no additional case with septin multimer IgG reactivity was identified. No multiple sclerosis patient was tested positive on CBA. CBA: cell-based assay, IIFA: indirect immunofluorescence assay. NF155: Neurofascin155

References

1. Iv F, Martins CS, Castro-Linares G, et al. Insights into animal septins using recombinant human septin octamers with distinct SEPT9 isoforms. *J Cell Sci*. 2021;134(15):jcs258484. doi:10.1242/jcs.258484

2. Miske R, Scharf M, Borowski K, et al. Septin-3 autoimmunity in patients with paraneoplastic cerebellar ataxia. *J Neuroinflammation*. 2023;20(1):88. doi:10.1186/s12974-023-02718-9

3. Arlt FA, Miske R, Machule ML, et al. KCNA2 IgG autoimmunity in neuropsychiatric diseases. *Brain Behav Immun*. 2024;117:399-411. doi:10.1016/j.bbi.2024.01.220

4. Gilligan M, Lesnick CE, Guo Y, et al. Paraneoplastic calmodulin kinase-like vesicle-associated protein (CAMKV) Autoimmune Encephalitis. *Ann Neurol*. 2024;96(1):21-33. doi:10.1002/ana.26943

5. Gilligan M, Mills JR, Vargas P, et al. Insights Into the Antigenic Repertoire of Unclassified Synaptic Antibodies. *Ann Clin Transl Neurol*. Published online November 6, 2025. doi:10.1002/acn3.70238

6. Vorasoot N, Scharf M, Miske R, et al. CDR2 and CDR2L line blot performance in PCA-1/anti-Yo paraneoplastic autoimmunity. *Front Immunol*. 2023;14:1265797. doi:10.3389/fimmu.2023.1265797
